# Proteomic Profiling of Intra-Islet Features Reveals Substructure-Specific Protein Signatures

**DOI:** 10.1016/j.mcpro.2022.100426

**Published:** 2022-10-14

**Authors:** Adam C. Swensen, Dušan Veličković, Sarah M. Williams, Ronald J. Moore, Le Z. Day, Sherry Niessen, Sarah Hennessy, Camilo Posso, Mara Monetti, Wei-Jun Qian, Jon Jacobs, Laurence Whiteley, Ying Zhu, Paul D. Piehowski

**Affiliations:** 1 Biological Sciences Division, Pacific Northwest National Laboratory, Richland, Washington, USA; 2 Environmental Molecular Sciences Laboratory, Pacific Northwest National Laboratory, Richland, Washington, USA; 3 Belharra Therapeutics San Diego County, California, USA; 4 Pfizer Worldwide Research and Development, La Jolla, California, USA; 5 Pfizer Worldwide Research and Development, Cambridge, Massachusetts, USA

**Keywords:** pancreatic islet, laser capture microdissection, nanoPOTS, intra-islet, microvasculature, drug-target interaction, ABC, ammonium bicarbonate, FAIMS, high-field asymmetric waveform ion mobility spectrometry, LCM, laser capture microdissection, nanoPOTS, nanodroplet processing in one-pot for trace samples

## Abstract

Despite their diminutive size, islets of Langerhans play a large role in maintaining systemic energy balance in the body. New technologies have enabled us to go from studying the whole pancreas to isolated whole islets, to partial islet sections, and now to islet substructures isolated from within the islet. Using a microfluidic nanodroplet-based proteomics platform coupled with laser capture microdissection and field asymmetric waveform ion mobility spectrometry, we present an in-depth investigation of protein profiles specific to features within the islet. These features include the islet-acinar interface vascular tissue, inner islet vasculature, isolated endocrine cells, whole islet with vasculature, and acinar tissue from around the islet. Compared to interface vasculature, unique protein signatures observed in the inner vasculature indicate increased innervation and intra-islet neuron-like crosstalk. We also demonstrate the utility of these data for identifying localized structure-specific drug–target interactions using existing protein/drug binding databases.

## Highlights

• We differentiated intra-islet substructures by their proteome profiles *in situ*.
• We demonstrated significant functional differences between vasculature types.
• Novel differences in innervation and colocalization of nerves were observed.
• We demonstrated a depth of coverage useful for predictive binding discovery assays.
• We identified pathways with these techniques that could aid drug characterizations.

## Graphical Abstract

**Figure:**
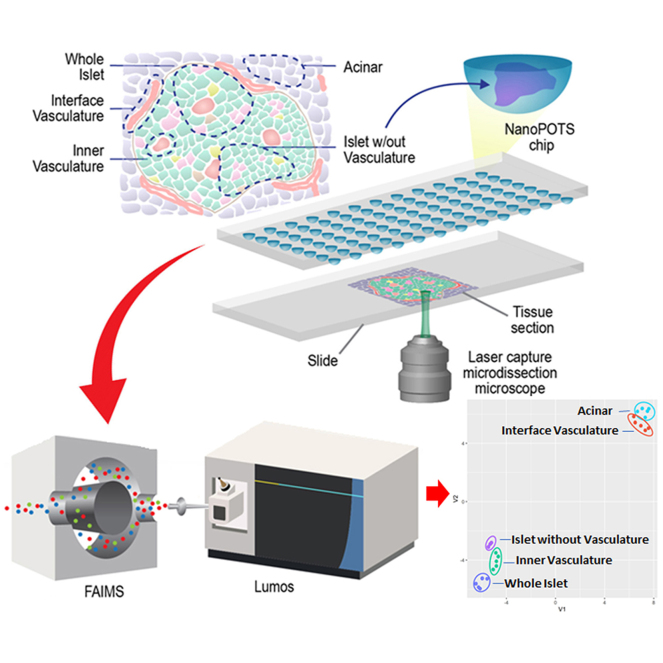

## In Brief

Improved sample handling techniques have enabled us to go from studying the whole pancreas, to isolated whole islets, to islet sections, and now to islet substructures isolated from within islet sections. Using a microfluidic nanodroplet-based proteomics platform coupled with laser capture microdissection and field asymmetric waveform ion mobility spectrometry, we present an in-depth investigation of protein profiles from intra-islet features. The protein coverage and expression profiles obtained are sufficient to fully distinguish each of these features while presenting their own unique proteome signatures.

Although small in size and collectively only comprising roughly 0.005% of the total body mass of an average adult (1), the islets of Langerhans are vital for whole-body survival, metabolism, energy balance, and appetite control. Every cell type in the body experiences some sort of impact from the endocrine islets. Despite their importance, there are significant knowledge gaps toward the comprehensive understanding of islets. New research continues to highlight the complex heterogeneity that exists in the islets of Langerhans within the pancreas (2, 3, 4); as we now know that their vascularization (5, 6), pancreatic sublocalization (7), and size, all impact islet function (8, 9). Such heterogeneity extends deeper than just the level of the whole islet. Recent research has shown that heterogeneity exists even within islet cell types; where at least four distinct β-cell phenotypes have been observed (10). Whole-islet phenotype and survival has also been shown to be affected by differences in vascularization within and surrounding the islets (11, 12). Cutting-edge technologies have even demonstrated that there appear to be hybrid cell types within and surrounding islets which express both exocrine and endocrine markers (13). It is no wonder that despite decades of research, we continue to reveal novel discoveries about these important micro-organs. This highlights the need to move islet research to a deeper level and to profile intra-islet heterogeneities with high spatial resolution.

State-of-art technologies can profile and image intra-islet features down to single-cell resolution through transcriptomics (3, 13, 14, 15, 16, 17, 18), ablative mass spectrometry imaging (19), or CyTOF (13). However, technologies to profile global protein-level signatures at such a small scale, with biologically significant depth-of-coverage, are still in their infancy. In an ideal scenario, a single-cell sample would yield full proteomic depth-of-coverage, sufficient to investigate all the proteins and pathways of interest. In practice, concessions must be made depending on the amount of sample available. As sample sizes become smaller, it becomes harder to achieve the concentration thresholds required for detection using existing instrumentation, hindering identification and quantification of low abundance proteins. When more sample mass is used, these concentration thresholds are more easily achieved, and the depth-of-coverage of the proteome increases. For this reason, most studies have used larger sample sizes (*e.g.*, 1000 islet equivalents) with multiple pooled islets to achieve adequate depth-of-coverage (20, 21, 22). Several techniques have been able to achieve good results at the single islet level (23, 24), but no attempts have been made at *in situ* profiling of intra-islet features.

The pancreatic islet and the associated microvasculature are occasionally the location of drug-induced injury, and understanding the biology of the microvasculature and potential interactions with adjacent components of the islet and acinar tissue may provide insight into mechanisms of injury and interspecies translation (25). The other potential impact for sub-islet proteomics study is for drug–protein interaction discovery. As is often the case in studies involving islets, it is difficult to identify specific drug–protein interactions when the typical adverse response is a complex whole islet state of inflammation. By narrowing the proteome signatures to specific features within the islet, we can significantly reduce the complexity of the system. By necessity, this depends on the ability to process low amounts of sample without significantly compromising the necessary depth-of-coverage to observe the proteins of interest.

**Fig. 1:**
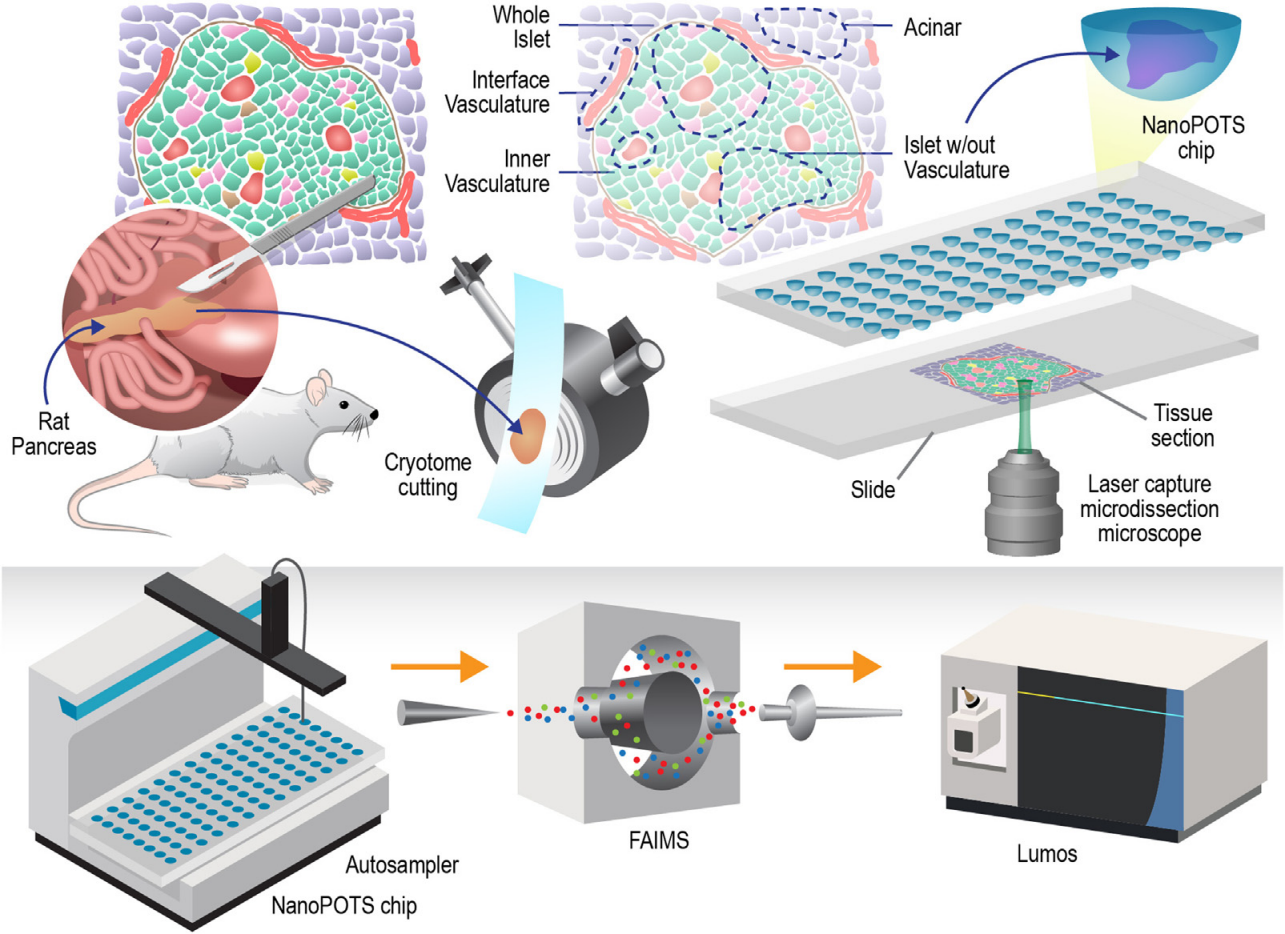
**An overview of the experimental plan from collection of the pancreas, slicing, selective microstructure selection, laser capture microdissection, and nanoPOTS preparation; to injection through LC separation and FAIMS to the mass spectrometer for data collection.** FAIMS, high-field asymmetric waveform ion mobility spectrometry; nanoPOTS, nanodroplet processing in one-pot for trace samples

Toward a better understanding of how islet heterogeneity can be measured at the protein level and to investigate how islets respond to external chemical stressors, we conducted proteomics analysis of distinct intra-islet microstructures of rat islets. The highly sensitive measurement is enabled by our recently developed nanoPOTS (nanodroplet processing in one-pot for trace samples) technology (23, 26, 27) (Fig. 1). NanoPOTS significantly improves proteomics sensitivity by reducing the sample preparation volumes to <200 nl. We demonstrated that reaction miniaturization can dramatically improve sample recovery and enhance protein digestion kinetics. In a previous study, we showed nanoPOTS were able to quantify ∼2400 proteins from single human pancreatic islet thin sections (23). In this study, we further improved the overall sensitivity of nanoPOTS proteomics platform by coupling it with a high-field asymmetric waveform ion mobility spectrometry (FAIMS) (28). The improved sensitivity enabled in-depth proteomic profiling of intra-islet substructures. These structures were isolated by laser capture microdissection (LCM), with each sample having an average area of 0.01 mm^2^ containing less than 100 cells.

We studied five distinct features within rat islets (25). These features (supplemental Figs. S1 and S2) included the outer-sheath endothelial vasculature that envelops the islet (*i.e.*, interface vasculature), the vasculature that perfuses the interior islet matrix (*i.e.*, inner vasculature), the interior islet tissue devoid of vasculature (*i.e.*, islet without vasculature), a wide all-encompassing section of islet which included all structures previously mentioned (*i.e.*, whole islet), and tissue external to the islet in the exocrine pancreas comprised mainly of acinar cells and associated microvasculature (*i.e.*, acinar). We explored the significant pathways enriched in each sub-islet feature and discussed how these could be potentially affected by a typical drug treatment such as a cysteine-binding inhibitor.

## Experimental Procedures

### Experimental Design and Statistical Rationale

The primary purpose of our experiments was to increase depth of proteome coverage in ultra-small spatially resolved samples with the intent to differentiate features at this scale. All sample for this experiment were collected from a single animal fresh frozen unstained primary tissue chunk across several 12 μm thin cryotome-sliced sections from the pancreatic tail. Each sample type was collected in pools with five technical replicates to allow for robust ANOVA comparison across the spatially separated microstructure types. Five replicates allowed for sample processing failures while still maintaining at least three comparative points for quantitation. Additional mixed model statistical tools were also applied to the data for data analysis and validation. The differences reported are in a single 7- to 8-week-old male rat pancreas without the complications of biological/sex differences between individuals.

### Collection of Pancreata From Rats and Imaging of Pancreatic Islet Vasculature

Sprague Dawley rats (Crl:CD[SD]) were obtained from Charles River Laboratories and were 7 to 8 weeks of age when pancreas was collected. Animals were euthanized by exsanguination while under isoflurane gas anesthesia. In order to label the endothelium of the pancreatic vasculature to guide collection with the LCM, a lectin cocktail (1 mg/ml or each lectin in phosphate buffered saline) composed of fluorescein-labeled *Lycopersicon esculentum* (Tomato, (TL)) (Vector Laboratories FL-1171–1) and Alexa-594-labeled wheat germ agglutinin (Thermo Fisher, W11262) was administered by the intravenous route through the jugular vein (10 ml/kg). Rats were euthanized 5 min after lectin injection, and the pancreas was collected immediately and snap-frozen in optimal cutting temperature compound. To also visualize vascular wall pericytes and smooth muscle, 10 micron cryosections were cut and labeled with a mouse monoclonal antibody to α-smooth muscle actin (1:500 dilution, R&D Systems clone 1A4) followed by Alexa-647-conjugated Donkey anti-mouse IgG (Jackson Laboratories 715–605–151). Sections were stained with 4′,6-diamidino-2-phenylindole and scanned with Zeiss imager. This staining confirmed the location of key vascular compartments of the islet (supplemental Fig. S1*A*). For visualization on the LCM, the a-SMA antibody and 4′,6-diamidino-2-phenylindole were not used. Only TL-fluorescein isothiocyanate and wheat germ agglutinin–Alexa-594 were imaged (supplemental Fig. S1*B*). After imaging the vascular structures with these techniques, it was discovered that endogenous tissue contrast, when sections were viewed with bright field illumination on the LCM microscope, enabled the vascular profiles and islet borders to be readily visualized (supplemental Fig. S1*C*). All LCM collections were done with bright field illumination. All animal handling procedures were approved by Pfizer’s animal care and use committee.

### Cryosectioning

Twelve micrometer-thick pancreatic tissue slices from rat pancreata were cut from frozen blocks using a cryo-microtome (Thermo Fisher, Cryostar NX70) with the specimen and blade kept at or below −20 °C. The thin sections were thaw-mounted on PEN membrane slides (Membrane Slide NF 1.0, ZEISS) and stored inside a cold desiccator until all sections were collected. Slides were then washed and fixed in 50% EtOH (2 min), 100%EtOH (1 min), xylene (2 min), and 100% EtOH (1 min). Slides were stored at −80 °C until ready for LCM.

### Laser Capture Microdissection

LCM was performed using a PALM MicroBeam LCM microscope (Carl Zeiss Micro Imaging GmbH) containing a RoboStage for high-throughput sample collection and a PALM RoboMover (PALM Robo software, version 4.9). Islet features were first observed with and without fluorescence activation using stained tissues to identify cell nuclei, outer vasculature endothelium, and inner vasculature endothelium, respectively (supplemental Fig. S2). The LCM cuts for the final experiment were completed using unstained pancreata sections guided by the similarity to morphological structures that were observed in the stained samples and easily distinguished without fluorescent markers based on the tissue contrast observed in bright field transmitted light illuminated sections. Dissections were performed under a 20× objective. Typical settings used for laser cutting were UV-Energy of 40 and UV-Focus of 67.

NanoPOTS chip with 1.4 mm-diameter nanowells were used for tissue collection. Prior to LCM, each nanowell was preloaded with a 250 nl dimethyl sulfoxide (DMSO) droplet for tissue capture. Cut elements were catapulted into the DMSO droplets, deposited on nanowells (26, 27) using the “CenterRoboLPC” function with an energy level of delta 22 and a focus level of delta 5. After dissection, samples were stored at −80 °C until ready for sample processing. Each LCM-cut tissue has an area of ∼10,000 μm^2^, and five replicates for each feature/substructure were collected. Except in the case of islets without the inner microvasculature where only three replicates were evaluated, due to capture issues where we were unable to verify section capture on the nanoPOTS chip. Also, for vasculature samples, the sample were comprised of multiple cut sections that were pooled from across several islets in the sample cryotome section in order to have an equivalent amount of protein available for the subsequent nanoPOTS sample preparation.

### NanoPOTS-Based Protein Extraction and Digestion

Before sample preparation, DMSO was evaporated by heating the nanoPOTS chips at 70 °C for 15 min. For cell lysis and protein extraction, 150 nl of cell lysis buffer containing 0.1% DDM and 2 mM dithiothreitol in a 50 mM ammonium bicarbonate (ABC) and 0.5× PBS buffer was added into each nanowell. The nanoPOTS chip is sealed with a cover and subsequently incubated at 70 °C for 60 min to lyse the cells, extract, and reduce the proteins. The protein sulfhydryl groups were alkylated by adding 50 nl of iodoacetamide solution (20 mM in 50 mM ABC) for 30 min at room temperature in the dark. A predigestion step was completed using 50 nl enzyme solution containing 0.5 ng Lys-C in 50 mM ABC at 37 °C for 4 h. For the primary digestion, 50 nl of enzyme solution containing 1 ng trypsin in 50 mM ABC was added to each droplet and incubated overnight at 37 °C. To stop the digestion, 50 nl of formic acid solution (5%, v/v) was added and incubated for 15 min at room temperature. To minimize liquid evaporation in nanowells, the robotic handling system was humidity controlled and the chip was completely sealed during incubation and transfer procedures. During each dispensing step, the chip was opened and closed within the humidity chamber to minimize droplet evaporation. However, some evaporative losses occurred, because the final volume was <200 nl despite adding 300 nl to each well during the preparation. These losses do not appear to degrade the overall experimental results. Finally, the droplets were completely dried for 15 min in a vacuum desiccator. The chip was wrapped in aluminum foil and stored in −20 °C freezer before LC-MS analysis.

### LC-MS

MS data collection was performed using a home-built nanoPOTS autosampler (29) and an Orbitrap Fusion Lumos Tribrid MS (ThermoFisher) with a FAIMS interface (30, 31). The LC separation system consisted of a SPE column [100 μm i.d., 4 cm, packed with 5 μm, C18 packing material (300 Å pore size; Phenomenex)] and a 50 μm, 60 cm long LC column packed with 1.7 μm, C18 packing material (BEH 130 Å pore size; Waters). The separation flow rate was 100 nl/min. A 120-min linear gradient from 8% Buffer B (0.1%FA in 100% ACN) to 30% Buffer B was applied for the peptide separation. Electrospray voltage of 2.6 kV was applied at the FAIMS source to ionize the peptides. The ion transfer tube was set at 200 °C for desolvation. Ion funnel radio frequency level was set at 30. Three FAIMS CVs (−45V, −60V, −75V) were used. At each CV setting, a full MS scan range of 350 to 1600 and Orbitrap resolution of 120,000 (at m/z 200) were used. The AGC target and maximum injection time were set at 1E6 and 118 ms, respectively. Data-dependent acquisition mode was used to trigger precursor isolation and sequencing. The signal intensity threshold was set at 1E4. Precursor ions with charges of +2 to +7 were isolated with an m/z window of 1.4 and fragmented by high energy dissociation with a collision energy of 30%. To minimize repeated sequencing, dynamic exclusion with a duration of 30 s and mass tolerance of ±10 ppm was utilized. MS/MS scans were performed in the ion trap with an AGC of 2E4 and a maximum injection time of 150 ms.

### Data Analysis

All raw files were processed using Maxquant (version 1.6.2.10) (32) for feature detection, database searching, and protein/peptide quantification using TIFF method as we described previously (28). The MS raw files were split into multiple files based on individual CVs (−45, −60, −75) using Thermo Scientific Xcalibur FreeStyle software (v1.7). When importing into MaxQuant, the MS raw files with different CVs were treated as different LC fractions; CVs of −45, −60, −75 were treated as fractions of 1, 3, 5. MS/MS spectra were searched against the UniProtKB/Swiss-Prot rat database (downloaded on April 10, 2018, 8020 unique proteins). N-terminal protein acetylation and methionine oxidation were selected as variable modifications. Carbamidomethylation of cysteine residues was set as a fixed modification. The peptide mass tolerance for the first search and recalibrated main search were <20 and 4.5 ppm, respectively. The match tolerance, *de novo* tolerance, and deisotoping tolerance for MS/MS search were 20, 10, and 7 ppm, respectively. The minimum peptide length was seven amino acids, and the maximum peptide mass was 4600 Da. The number of allowed missed cleavages for each peptide was 2. The second peptide search was activated to identify co-eluting and co-fragmented peptides from one MS/MS spectrum. Both peptides and proteins were filtered with a maximum false discovery rate of 1%. The match between runs (MBR) feature, with a match window of 0.7 min and an alignment window of 20 min, was activated to increase peptide/protein identification. Label-free quantification calculations were performed separately in each parameter group containing similar LCM surface area loading from each substructure. Both unique and razor peptides were selected for protein quantification. Requiring MS/MS for label-free quantification comparisons was not activated to increase the quantifiable proteins. Any unmentioned parameters were set to the default MaxQuant software settings.

Statistical analysis was completed and visualized using Microsoft Excel and the in-house developed R statistics program InfernoRDN (v1.1.7234) (33). Gene ontology analysis was conducted using DAVID (34) and visualized using GOrilla (35).

## Results

### Depth of Proteome Coverage

**Fig. 2:**
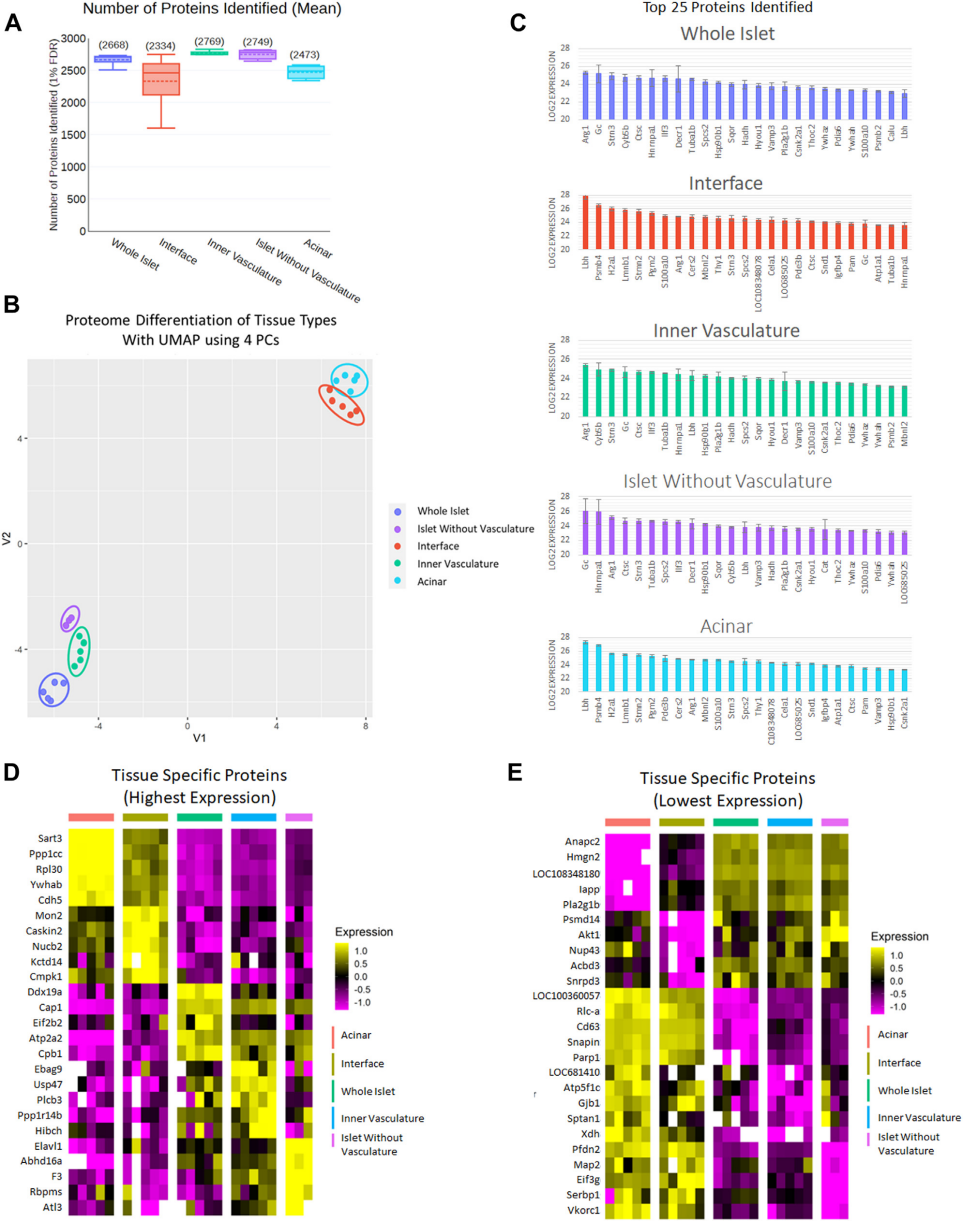
**Overall depth of coverage, substructure differentiation, and proteins with the highest and most significant expression.***A*, the depth-of-coverage achieved during this study. A total of 3079 unique proteins were identified across all feature types. The graph represents how many of those unique proteins were identified in each feature type. *B*, differentiation of islet microstructures based on global proteomics using UMAP with four principal components (PCs). *C*, top 25 proteins per tissue type based on absolute abundance. *D*, heatmap showing proteins with statistically significant expression differences in the target tissue and the highest expression levels. *E*, heatmap showing proteins with statistically significant expression differences in the target tissue with the lowest detectable expression in each of the tissue types.

The protein abundance profiles for individual intra-islet substructures are not reported previously. According to the Human Protein Atlas transcriptome analysis, 74% (n = 14,490) of all theoretical human proteins (n = 19,670) are expressed across the entire pancreas (36, 37). A later transcriptomics study observed 9804 nonalternately spliced transcripts in isolated islets (38). Using a large, pooled sample of whole islets (*i.e.*, >1000 islet equivalents), MS-based proteomics study was able to observe 6873 total proteins (24). This study is the first-of-its-kind proteomics study of *in situ* intra-islet microstructures. Using our recently developed nanoPOTS-based ultrasensitive proteomics platform, we aimed to sample intra-islet features corresponding to <0.1 islet equivalents without compromising sensitivity and coverage (Fig. 1). Using pooled LCM cuts, we achieved coverage sufficient to differentiate each tissue type sampled. Using a false discovery rate cutoff of <1% at both the peptide and protein level and only quantifying proteins containing at least two peptides, we were able to quantify 3066 total unique proteins (protein groups), with average quantifications of 2473 proteins in acinar (exocrine pancreas), 2334 proteins in interface vasculature, 2668 proteins in whole islets, 2749 proteins in islets without microvasculature, and 2769 proteins in the inner microvasculature (Fig. 2*A*). These proteins were determined from 17,394 unique peptides. Average peptide identification by features were 11,627 in acinar, 10,574 in interface vasculature, 12,411 in whole islets, 13,427 in inner microvasculature, and 13,264 in islets without microvasculature.

### Distinct Global Proteome Profiles

**Fig. 3:**
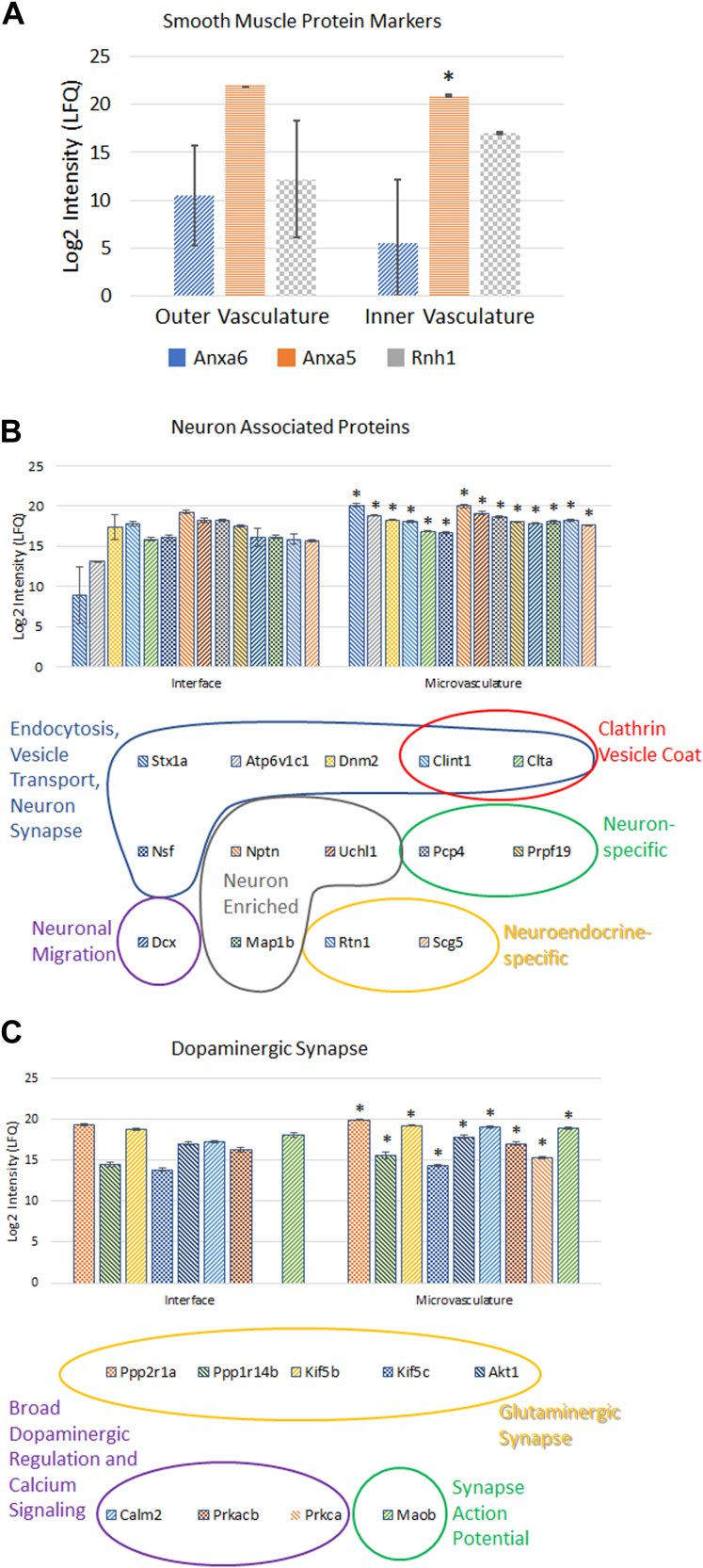
**Differences between inner microvasculature and surrounding interface vasculature.***A*, smooth muscle marker proteins were expressed at similar expression levels in the inner microvasculature when compared to the vasculature interface outside of the islet. (∗*p* < 0.05, Student’s 2-tailed paired *t* test) (Special note: while these proteins are known to be associated with smooth muscle, their expression is not specific and expression can be observed in other tissues, *i.e.*, Anxa6 can be found in skeletal muscle and cell membranes within the islet). *B*, plot of neuron-associated proteins detected in this study and their respective label-free quantification (LFQ) in each type of vasculature. (∗*p* < 0.05, Student’s 2-tailed homoscedastic *t* test). *C*, plot of dopaminergic synapse proteins detected in this study and their respective LFQ in each type of vasculature. (∗*p* < 0.05, Student’s 2-tailed homoscedastic *t* test).

We generated proteome profiles of spatially resolved sub-islet features that identified the most abundant and least abundant proteins unique to each feature (Fig. 2, *C* and *D*). As we anticipated, each of the feature types did have varying amounts of contaminating protein signals from the neighboring tissue types, due to the imperfect laser dissection steps. To control for this, we included a set of “whole islet” samples which contained both outer interface vasculature and inner microvasculature, which we could compare to the “pure” feature samples (acinar, vasculature, and islet), to enhance our confidence that the protein was specific to that particular feature by comparative quantitative analysis. Each of the five sampled features was sufficiently distinct in their protein profiles to be fully resolved by UMAP clustering (Fig. 2*B*). As expected, these separations were most significant when differentiating exocrine (acinar) *versus* endocrine (islet) proteome profiles. We also confirmed the separations were not solely based on highly abundant islet hormones acinar enzymes and vasculature proteins (supplemental Fig. S3). The additional features incorporated by the UMAP analysis improve differentiation, while identifying new potential tissue differences. However, it was surprising to note that the proteome profiles of the two islet containing features (whole islets and islets without inner microvasculature) and the two vasculature feature types (interface vasculature and inner microvasculature) separated completely. Important to note is that smooth muscle–associated vasculature-enriched proteins annexin A5 (Anxa5), annexin A6 (Anxa6) (39), and ribonuclease/angiogenesis inhibitor 1 (Rnh1) showed similar expression levels in the interface vasculature compared to the inner microvasculature (Fig. 3*A*), which helped to ensure that vasculature was being sampled. It deserves to be mentioned that Anxa6 in particular, while known to be associated with vasculature, is not solely expressed in vasculature and can be observed in skeletal muscle and cell membranes of pancreatic cells in addition to the enriched expression in vasculature. More biologically significant differences were observed, especially between vasculature types, which we will discuss later in greater detail.

### Feature-Specific Protein Markers

We first evaluated if tissue-specific markers could be observed in their respective tissue substructures. While it would be unrealistic to assume that all the samples were collected without contamination from neighboring features, we did still observe increased expression of known tissue-specific markers (Fig. 2, *C* and *D*). Though there were five different sample types analyzed, there were three major related features being profiled. The groupings were (1) exocrine pancreas primarily composed of **acinar** cells; (2) endothelial-lined **vasculature** (observed in both the outer vasculature and the inner microvasculature), and (3) endocrine interior **islet** (which includes the five hormone-producing cell types specific to islets, α cells, β cells, δ cells, ε cells, and PP cells).

As expected, the ”cleanest” signal originated from the **acinar** LCM cuts because these were collected from regions with greater spatial clearance from the other tissue types. It is worth mentioning that these cuts are expected to contain some unvisualized periacinar endothelium. This is demonstrated by the low-level detection of vasculature markers. However, no endocrine markers were detected. Proteins specific to acinar tissue are primarily secreted enzymes that aid digestion, such as trypsin (Try5, Tryb, Prss1, PRss3b), chymotrypsin-like elastases (Cela2a, Cela1, Cela3b, Ctrl, Ctrb1), lipases (Pnlip, Pnliprp1, Pnliprp2), and amylase (Amy2a3). Due to the nature of these proteins being secreted, it was expected to observe many of these proteins in the vasculature, where they would normally be secreted. Additionally, blood-specific proteins (*e.g.*, albumins, globulins, etc.) were enriched in vasculature as was observed in previous studies looking at larger bulk tissue samplings (23)(Data not shown). As expected, we observed many of these specific proteins among the most abundant proteins from the acinar samples with some of the most abundant proteins being pancreatic alpha-amylase (Amy2), pancreatic triacylglycerol lipase (Pnlip), alpha amylase (Amy1a), ribosome-binding protein 1 (Rrbp1), and protein disulfide isomerase (P4hb). The most unique proteins with expression levels higher in their respective substructure are shown in Fig. 2*C* (Sart3, Ppp1cc, Rpl30, Ywhab, Cdh5) and the most unique proteins with the lowest detectable concentrations in terms of the respective substructure are shown in Fig. 2*D* (Anapc2, Hmgn2, LOC108348180, Iapp, Plan2g1b).

The next feature group with similarity closest to the acinar samples were the **vasculature**. Due to the excretory nature of the pancreas, we expected significant levels of nonvasculature-originated contamination in these samples. For this reason, we decided to include additional samples with features that controlled for the inclusion of vasculature, such as the whole islets (which included inner microvasculature and interface vasculature in the cut sections) and islet tissue without identifiable microvasculature. This allowed us to distinguish vasculature-specific signatures across samples and control for contaminating signatures. Vascular protein markers observed in this group include nonmuscle myosin Heavy Chain 9 (Myh9), annexin a2 (Anxa2), annexin a11 (Anxa11), endothelial differentiation-related factor 1 (Edf1), and Septin 2 (Sept2). Several proteins with statistically unique high expression for inner microvasculature and interface vasculature are shown in Fig. 2*C* (Ebag9, Usp47, Plcb3, Ppp1r14b, and Hibch in inner microvasculature; and Mon2, Caskin2, Nucb2, Kcted14, and Cmpk1 in interface vasculature). Other highly abundant proteins in the isolated inner microvasculature were endoplasmic reticulum chaperone BiP (Hspa5) and hemoglobin (Hbb). The top most unique proteins in the inner microvasculature were probable E3 ubiquitin-protein ligase (Irf2bpl), arginine and glutamate-rich protein 1 (Arglu1), ENAH actin regulator (Enah), abl-interactor 2 (Abi2), and acetoacetyl-CoA synthetase (Aacs). The most abundant proteins in the interface vasculature were dominated heavily by acinar-specific proteins, including pancreatic alpha-amylase (Amy2), pancreatic triacylglycerol lipase (Pnlip), alpha amylase (Amy1a), Carboxypeptidase B (Cbp1), and ribosome-binding protein 1 (Rrbp1). The most unique proteins were also dominated by acinar-originated proteins including anionic trypsin 2(Prss2), secernin 2 (Scrn2), translocon-associated protein subunit gamma (SSr3), alpha-1,2-mannosidase (Man1a1), and trypsin B (TryB). The heavy contribution of acinar-specific proteins is likely due to the proximity of these isolations to the two anatomic compartments where the microvasculature traverses. The most unique detectable proteins with lowest expression are shown in Fig. 2*D* (LOC681410, Atp5f1c, Gjb1, Sptan1, and Xdh in inner microvasculature and Psmd14, Akt1, Nup43, Acbd3, and Snrpd3 for interface vasculature).

Finally, we looked at the endocrine-specific islet cells without vasculature. While we did see endocrine hormones that we were expecting, such as insulin (Ins1) and glucagon (Gcg) (predominantly in whole islets), we also observed a sizeable amount of what appears to be blood contamination in these samples as hemoglobin subunit beta-1 (Hbb) and hemoglobin subunit alpha-1/2 (Hba1) were two of the most abundant proteins detected. The structural protein cytoplasmic actin 1 (Actb) and the endoplasmic reticulum chaperone BiP (Hspa5) were among the most abundant proteins detected, although not as unique to the tissue as other proteins highlighted in Fig. 2, *C* and *D* (Abhd16a, F3, Rbpms, Atl3 for high expression; and Pfdn2, Map2, Eif3g, Serbp1, Vkork1 for lowest expression). The most unique proteins across **both** types of islet samples (with vasculature and without vasculature) were crystallin beta-gamma domain-containing 3 (Crybg3), inositol hexakisphosphate, and diphosphoinositol-pentakisphosphate kinase (Ppip5k2), an uncharacterized protein, Hsp70-binding protein 1 (Hspbp1), Ttll3 protein (Ttll3), protein arginine N-methyltransferase 5 (Prmt5), tropomodulin-1 (Tmod1), proteasome activator subunit 4 (Psme4), glycosylphosphatidylinositol anchor attachment 1 (Gpaa1), and glycophorin-C (Gypc).

### Enriched Signatures Characteristic of Innervation and Neuron-Vasculature-Islet Crosstalk Within the Islet Inner Vasculature

Intriguingly, our datasets revealed differences between the two vasculature types found in and around islets, the interface vasculature and inner microvasculature, after accounting for acinar and islet contamination. For instance, despite having nearly the same expected endothelial/vascular-specific marker expression as the interface vasculature, the inner microvasculature presented a statistically significant increase in neuron-associated proteins involved in synaptic junctions, neuron migration, and the glutaminergic synapse. Past studies have shown that parasympathetic ganglia have been associated with islets (40) and endothelial growth is known to recruit nerves (41). The peri-islet vasculature, or interface vasculature, has been hypothesized to be primarily used for drainage and therefore may not contain innervation like the trans-islet microvasculature, which we further confirm. Rat islets experience neural control through innervation by both sympathetic and parasympathetic autonomic nerves (42, 43, 44) and that innervation of islets appears to be important for survival and function (45), as well as being significant in diseases like type 1 and type 2 diabetes (42). Owing to the complexity, limited research has been conducted to show exactly how the internal innervation architecture is arranged (46). As mentioned, some evidence suggests that the nerves interact closely with vasculature and may influence vasculature during islet development. We observed several neuron-related pathways with statistically significant enrichment patterns in the islet inner microvasculature. The first pathway is the synaptic vesicle cycle, where we observed increased expression of proteins involved in vesicle formation, packing, and vesicle endocytosis; namely, vesicle associated membrane protein (Vamp), syntaxin (Stx1a), clathrin light chain A (CltA), clathrin interacting protein (Clint1), N-ethylmaleimide sensitive fusion protein (NSF), dynamin (Dnm2), and V-type proton ATPase subunit C 1 (V-ATPase) (Fig. 3*B*). There were also several proteins with significantly increased expression that are specific to neurons (Pcp4, Prpf19), enriched in neurons (Nptn, UchI1, Map1b), involved in neuronal migration (Dcx), or neuroendocrine specific (Rtn1, Scg5) as documented in the Human Protein Atlas (47). Another important neural crosstalk pathway we discovered to have increased expression in inner microvasculature is the dopaminergic synapse. Proteins we detected in our data were serine/threonine-protein phosphatase 2A 65 kDa regulatory subunit A (Ppp2r1a), kinesin-1 heavy chain (Kif5b), calmodulin 2 (Calm2), Monoamine oxidase B (Maob), protein kinase B (Akt1), cAMP-dependent protein kinase catalytic subunit beta (Prkacb), Circadian Locomotor Output Cycles Kaput (Clock), protein phosphatase 1 regulatory inhibitor subunit 14B (Ppp1r14b), kinesin family member 5C (Kif5c), and an important protein vital for tight-junction formation only detected in the inner microvasculature, protein kinase C alpha (Prkca) (Fig. 3*C*). We also observed several important proteins with expression differences involved in general endocytosis, which could also be involved in synapse-like activity (data not shown). Based on these findings, we hypothesize that internal innervation remains within proximity to the microvasculature owing to the enriched neuron-like protein signatures we observed in the microvasculature. The second hypothesis is that our data suggest that the microvasculature itself may be participating in limited neuron-like crosstalk with the autonomic nerves and/or the islet cells themselves. Evidence exists in the literature for the participation of microvasculature in neural regulation either through blood flow regulation or direct signaling in the brain (48, 49, 50) and heart (51). Human islets do not share the same highly innervated architecture that is observed in rodent islets (46), and the mechanisms of neural control in human islets remain unclear. The signaling observed in this study, namely the microvasculature-enriched L-Dopa dopaminergic pathway, may aid translational studies using human islets to better understand the complex microenvironmental controls at play in the pathogenesis of islet-related diseases. It has been suggested that localized vasoconstriction and inflammation of microvasculature through neural crosstalk could indirectly influence the development of autoimmunity in several diseases through immune cell trapping (52). Despite these exciting findings from the sub-islet proteomics, additional study with expanded species exploration and conditional variation is worthwhile. This vital information should help us to understand the contributions of islets and the sub-islet microstructures to disease etiology and pathogenesis.

### Ligandable Cysteine-–Containing Proteins Toward Drug–Target Interactome at the Sub-Islet Scale

The ability to probe the proteome of islet microstructures opens the door to many exciting new research directions. In addition to providing greater understanding for differentiation of regulatory pathways in proximal features, it could also be used to observe cell-to-cell communication, pinpoint protein–protein interactions, or determine specific drug–target interactions.

**Fig. 4:**
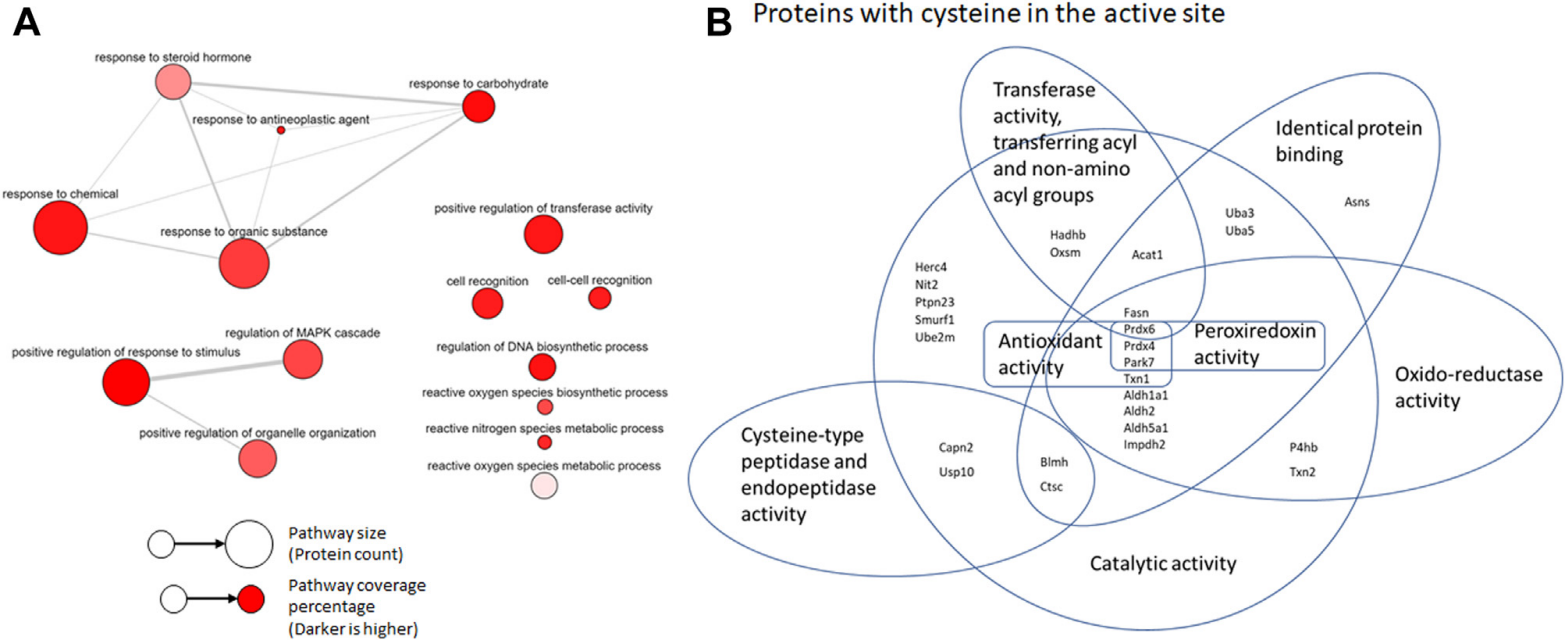
**Utility of the dataset to determine possible pathway perturbances and binding targets.***A*, diagram of significantly enriched pathways observed within the endocrine islet. *B*, a diagram of known cysteine-binding proteins detected and the biological processes in which they participate within the islet that could be affected by cysteine-targeted drug treatment.

Utilizing previously curated databases that annotate the presence and binding availability of cysteine amino acids in the active site of enzymes, we can predict where and how potential cysteine-binding drugs could interact within the islet. Compared with the surrounding acinar tissue, there is an enrichment in two major pathway clusters as well as some general pathways that could be affected in the presence of a cysteine-binding drug (Fig. 4*A*). The main enriched cluster of enzymes potentially affected by a cysteine-binding drug involved proteins involved in acyltransferase activity (including transferring groups other than amino-acyl groups)(Acat1, Hadhb, Prdx6, Fasn, and Oxsm) and in oxidoreductase activity (Prdx6, Impdh2, Txn1, Prdx4, P4hb, Aldh2, Txn2, Aldh1a1, Park7, Aldh5a1, and Fasn). The latter shows overlap with the closely related peroxiredoxin activity group of enzymes (Prdx6, Prdx4, and Park7) and antioxidant activity group of enzymes (Prdx6, Txn1, Prdx4, and Park7). Unsurprisingly, cysteine-type peptidase and endopeptidase enzymes (Blmh, Capn2, Usp10, Ctxc) were observed to be enriched in the islet (Fig. 4*B*). Other highly represented pathways that could be affected by potential cysteine-binding drugs included the general catalytic activity enzyme group. While more proteins could potentially be found with cysteine binding activity if the depth of coverage were increased, the proteins we did identify direct attention toward off-target effects from drug treatment in a region-specific manner.

### Limitations of the Current Study

Due to the nature of this study using limited available tissue from a single animal and due to the inherent design of the experiments to enhance the instrumental method development, the biological interpretation is limited to the data available. The biological interpretations herein serve as guide maps for additional study. Further validation studies would be required to confirm the biological interpretations presented herein. Additionally, the samples utilized in this study came from a single individual and therefore do not take into consideration the variability that could be observed due to age and/or sex. More interesting biological interpretation may be found upon further investigation of the data obtained from this study due to the depth of coverage and quality of the quantitative data.

## Discussion

Studies focused on the islets of Langerhans are inherently challenging. These tiny endocrine micro-organs are embedded within the mostly exocrine-containing pancreas and can be challenging to isolate and study without disrupting the complex tissue structure. Additionally, islets vary depending on their location within the pancreas and contain delicate internal features.

Identifying a small number of proteins and obtaining limited quantitative measurements from nanoscale samples is routinely attainable; however, obtaining global-scale measurements with depth-of-coverage that is useful at discovering biologically significant pathways is difficult. Traditional biochemical methods such as antibody-based ELISA assays and immunoprecipitation (IP) assays such as Western blots or IP-MS (or IP-SRM) can identify and quantify specific target proteins with small starting amounts. However, these methods often rely on prior knowledge to study the specific targets identified previously. Even when multiplexed, these methods only provide limited depth-of-coverage and are therefore not suitable tools for protein signature discovery. When cellular changes occur due to unknown mechanisms, it becomes necessary to identify proteins on a broader scale as we have demonstrated here at a sub-islet scale. Additionally, high-quality discovery datasets serve as a community resource for data mining due to unbiased data collection methods and broad protein coverage.

In this study, we employed our nanoPOTS-based ultrasensitive proteomics platform to study the islet microenvironment in an untargeted fashion. The coupling of nanoPOTS with LCM has allowed discovery studies of ever smaller and more precise regions within the pancreas, now with the implementation of FAIMS, within the islet itself. Early proteomics studies required larger starting amounts of tissue due to sample handling losses and instrument sensitivity. Instrument sensitivity has increased dramatically over recent years and has therefore placed more responsibility on the sample handling to increase depth-of-coverage regarding small starting sample amounts. The tradeoff to achieve even greater spatial resolution in tissues usually meant fewer protein identifications. With fewer protein identifications, the usefulness of the data decreases because important protein targets are no longer identified or quantified. Our goal is to maintain or improve useful depth-of-coverage while simultaneously utilizing smaller starting sample amounts, corresponding to greater spatial resolution of fine details. This is why proteomics studies have advanced from studying the pancreas as a whole, to pooled islets, to single islets, then to single islet sections, and to now obtaining useful depth-of-coverage on islet microstructures. We have demonstrated that our platform can reliably quantify ∼3000 proteins from <0.1 islet equivalents, which represents the deepest proteome coverage for islet microstructures. Outside of flow cytometry-based experiments in islets (17, 53, 54), no previous study has looked at global protein expression at sub-islet scales *in situ*.

As we have demonstrated in this study, the protein coverage and expression profiles are sufficient to fully distinguish each of the distinct features while presenting their own unique proteome signatures. Even though there is some measured cross-sample contamination due to the anatomical proximity of the structures during isolation, the data quality is sufficient to determine dominant expression patterns and unique tissue specific protein signatures. We demonstrated clear principal component separation of isolation types based on protein identification and abundance measurements. Tissue-specific proteins were detected in the acinar tissue samples and within the islet itself. Secreted proteins from both acinar and endocrine islet cells were detected in vasculature as expected due to the secretory nature and anatomical proximity of the vasculature to these tissues. Intriguingly, when controlling for the contribution of acinar/exocrine and islet/endocrine protein signatures within the vasculature signatures, we found differences between the vasculature that surrounds the islet and the microvasculature that traverses the interior of the islet. Signatures relating to possible increased innervation and neuron-like crosstalk, either with vasculature-following autonomic nerves or between the endothelial cells and the endocrine cell types, were detected in the microvasculature. While some of this signature undoubtably comes from the secretory contribution of the endocrine cells related to hormone secretion, the same levels were not detected in outer vasculature despite similar proximity to secretory cell types. This provides additional data to support the recent hypotheses that innervation and neuron-like endocrine-endothelial crosstalk with microvasculature may play significant roles in hormone regulation and response within the islet.

To further leverage the data, we have given an example of how these data could be used to look at region-specific drug–target effects with a hypothetical cysteine-binding drug treatment. Within the islet, there is an enrichment of acyltransferase, oxidoreductase, and peroxiredoxin enzymes that would be among the most highly affected pathways. This is in addition to the expected cysteine-type peptidase and endopeptidase activity enzymes and many broad category catalytic activity and identical protein binding activity enzymes. This demonstrates a proof-of-concept idea that these methods could be useful in determining drug–target effects for identifying pathways in a specific drug treatment experiment. This could be particularly useful for example, to identify off-target effects of a drug treatment specific to a feature or region within the islet or tissue being studied. Further, this demonstration illustrates the significant potential value of this dataset, and future datasets generated using this approach, as a data resource for the broader research community.

While it is not the focus of the current study, we expect the nanoPOTS-LCM platform can be extended to study individual single cells from islets. We have demonstrated the integration of isobaric labeling with nanoPOTS has enabled to study FACS-isolated single cells with a proteome coverage of ∼1500 proteins (55, 56, 57). The use of isobaric labels not only significantly increased the sensitivity but also improved the analysis throughput, as up to 16 samples can be measured with single LC-MS run. We envision that, with further development, it is possible to globally map the proteome inside islets at single-cell resolution to reveal the complexity of islet functions.

## Data Availability

Data are available through MassIVE, a full partner of ProteomeXchange, through the following database accession: MSV000088730 (https://massive.ucsd.edu)

## Supplemental data

This article contains supplemental data (58).

## Conflict of interest

The authors declare no competing interests.

## Supplemental Data

Supplemental Figures

Supplementary Tables Protein and Peptide Quant rev3

Single PSM protein spectra
