# Supplementary material for: Proteomic Profiling of Intra-Islet Features Reveals Substructure-Specific Protein Signatures
Source: Mol Cell Proteomics. 2022 Oct 14;21(12):100426. doi: 10.1016/j.mcpro.2022.100426 (PMC9706166; doi:10.1016/j.mcpro.2022.100426)
Supplement: Supplemental Figures [file mmc1.docx]

Supplemental Figures


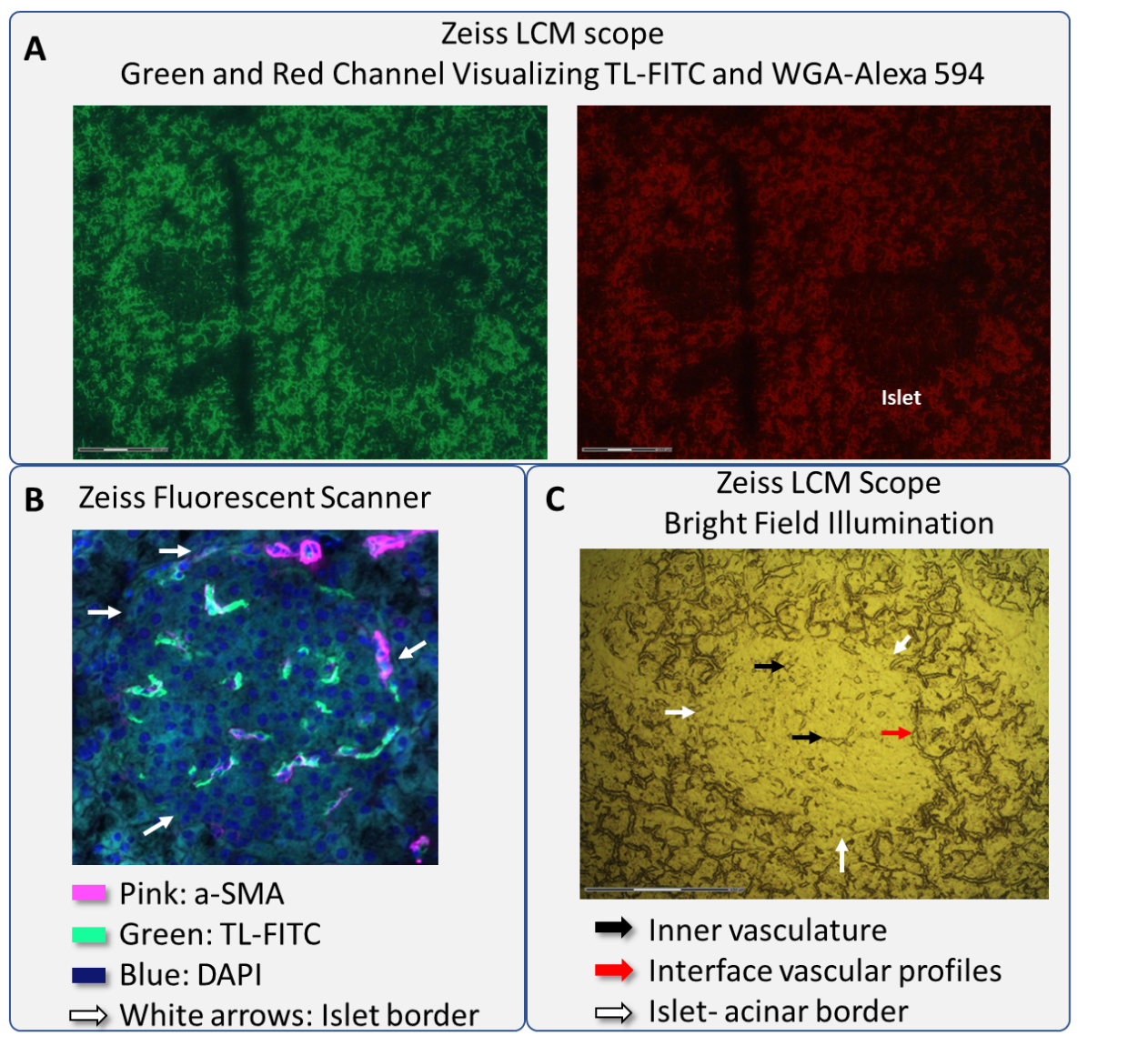


**Supplemental Figure 1:** Stained tissue was used to guide and instruct the LCM cuts on unstained sections. Tissues were stained with DAPI nuclear stain (to identify nuclei, blue), Tomato lectin + WGA 594 (nRFP) (to identify endothelium belonging to larger vasculature found outside of the islet, pink), and Tomato lectin + FITC (GFP) (to identify endothelium belonging to the inner microvasculature of the islet, green). Stained sections were observed in both excited and non-excited fluorescence modes to visualize and confirm what intra-islet features looked like prior to cutting the unstained sections. (Note: A) Represents imaging of the same section. B) and C) do not represent the same islet being imaged.)


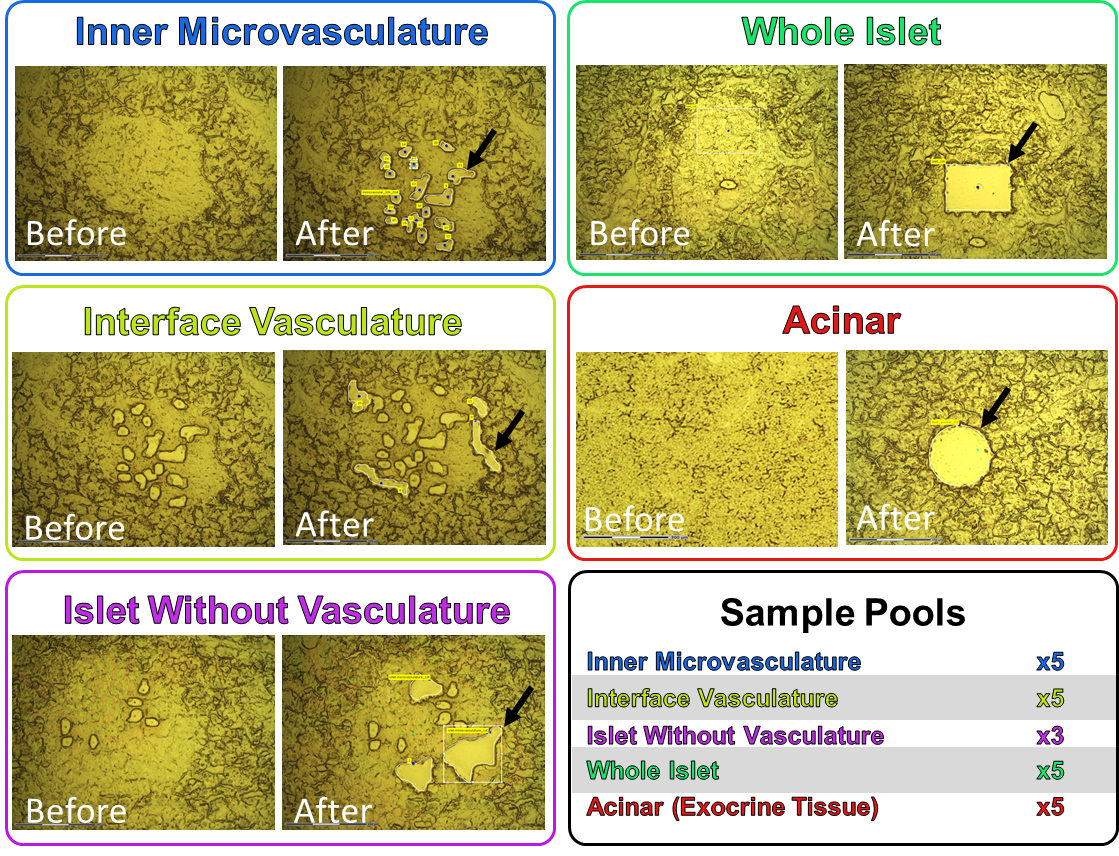


**Supplemental Figure 2:** Laser capture microdissection (LCM) before and after cuts of representative intra-islet features. For the small features, such as the vasculature, multiple small LCM cuts were pooled across the islet to equalize starting protein amounts more closely to the other respective feature cuts. Each feature was captured in 5x replicates, except for islets without vasculature where feature capture failed for two cuts.


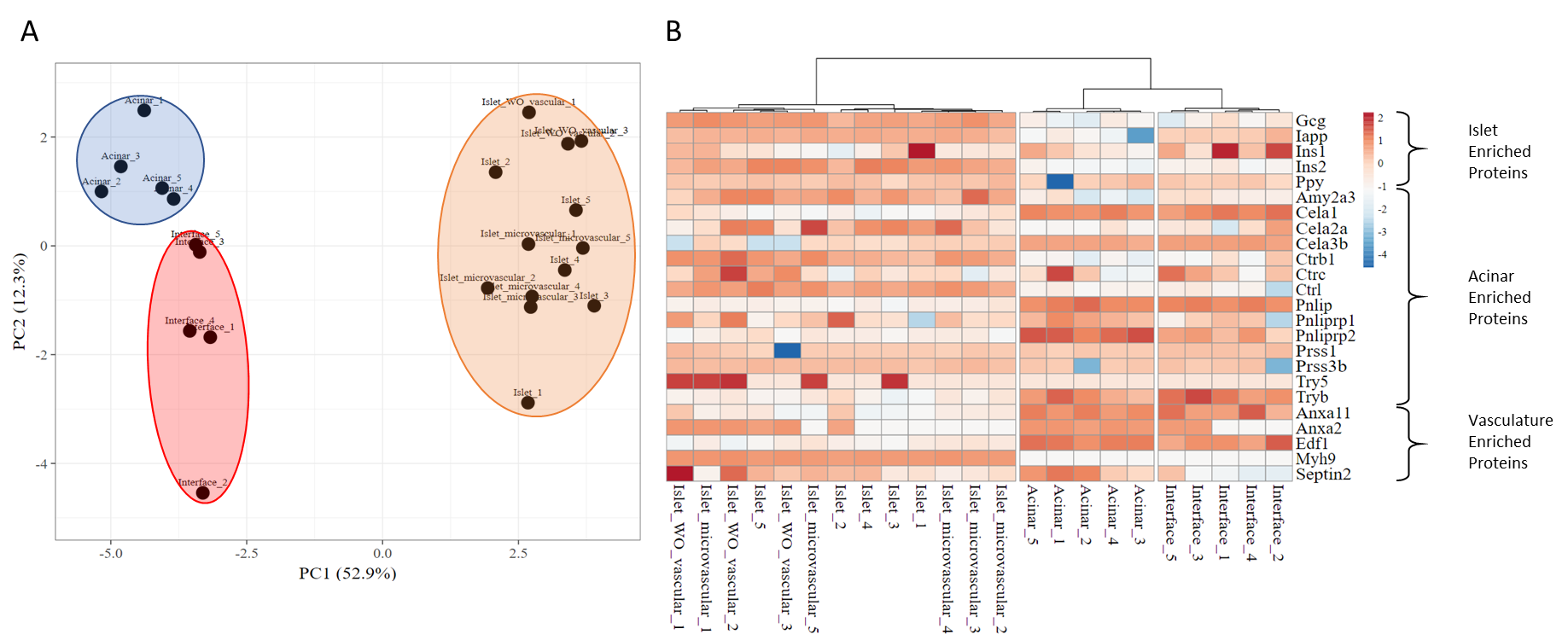


**Supplemental Figure 3:** Separation of regional features based on enriched tissue type protein markers. The five regional features mentioned throughout the study comprise primarily of 3 distrinct cell population types, acinar, vasculature, and islet. Using proteins markers expected to be enriched in each of these populations separation can be observed for the regionally separated cell populations both through PCA (A) and hierarchical heatmap clustering based on expression fold change (B). Of note here is the grouping of internalized islet features with the closest regional proximity.
