# Supplementary material for: Proteomic Profiling of Intra-Islet Features Reveals Substructure-Specific Protein Signatures
Source: Mol Cell Proteomics. 2022 Oct 14;21(12):100426. doi: 10.1016/j.mcpro.2022.100426 (PMC9706166; doi:10.1016/j.mcpro.2022.100426)
Supplement: Single PSM protein spectra [file mmc3.pptx]

## Slide 1
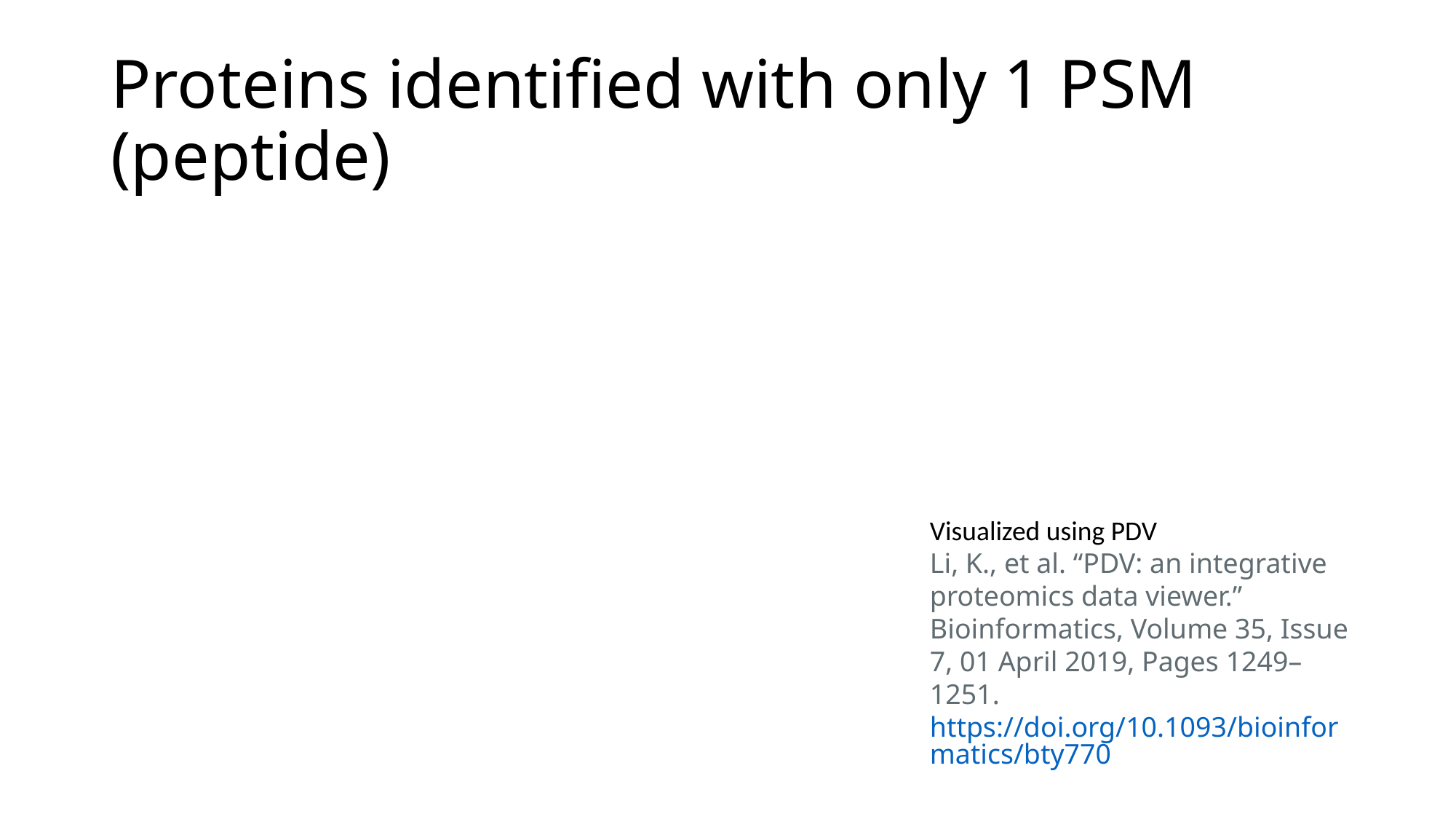

# Proteins identified with only 1 PSM (peptide)
Visualized using PDV
Li, K., et al. “PDV: an integrative proteomics data viewer.” Bioinformatics, Volume 35, Issue 7, 01 April 2019, Pages 1249–1251. https://doi.org/10.1093/bioinformatics/bty770

## Slide 2
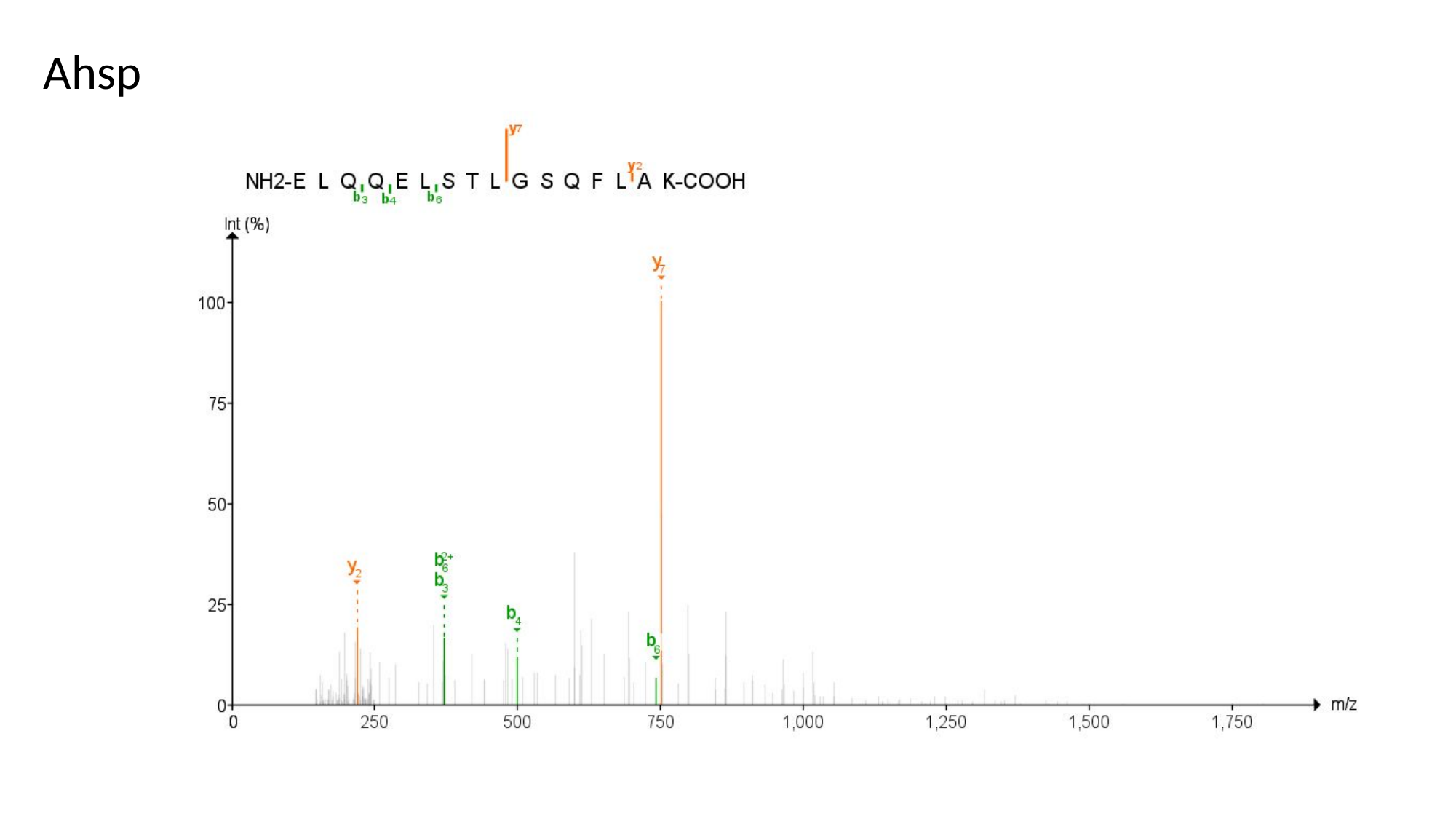

Ahsp

## Slide 3
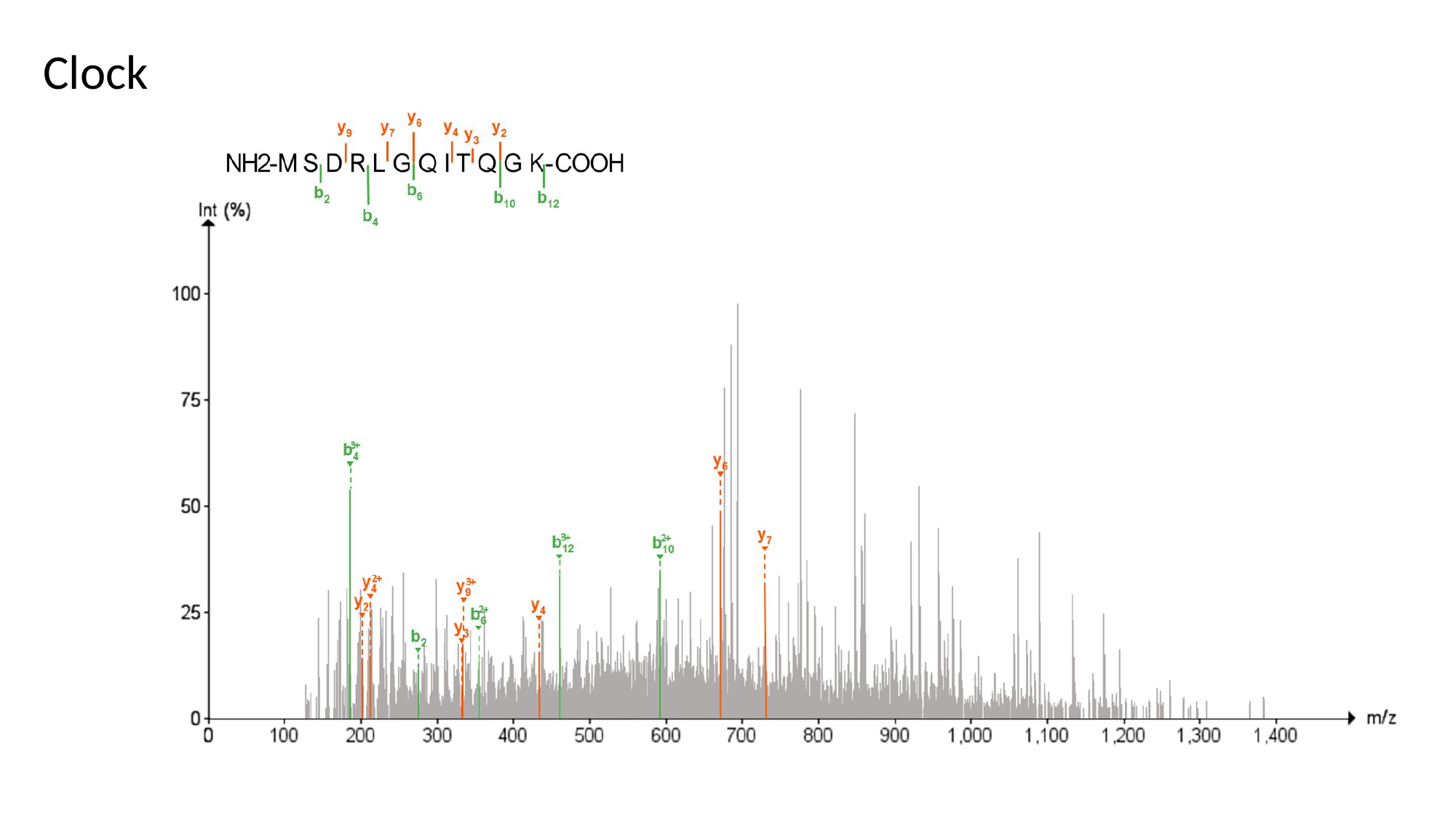

Clock

## Slide 4
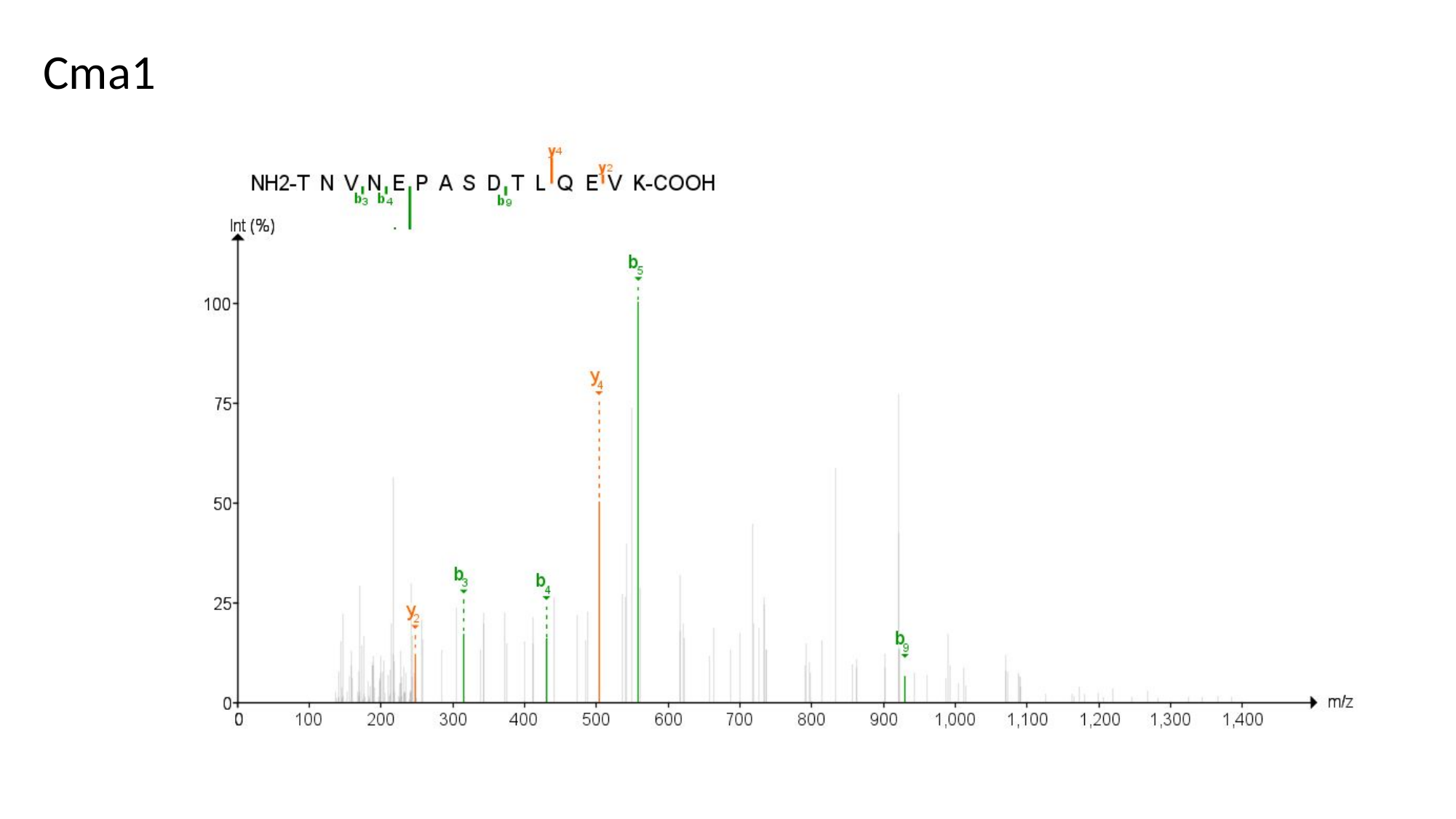

Cma1

## Slide 5
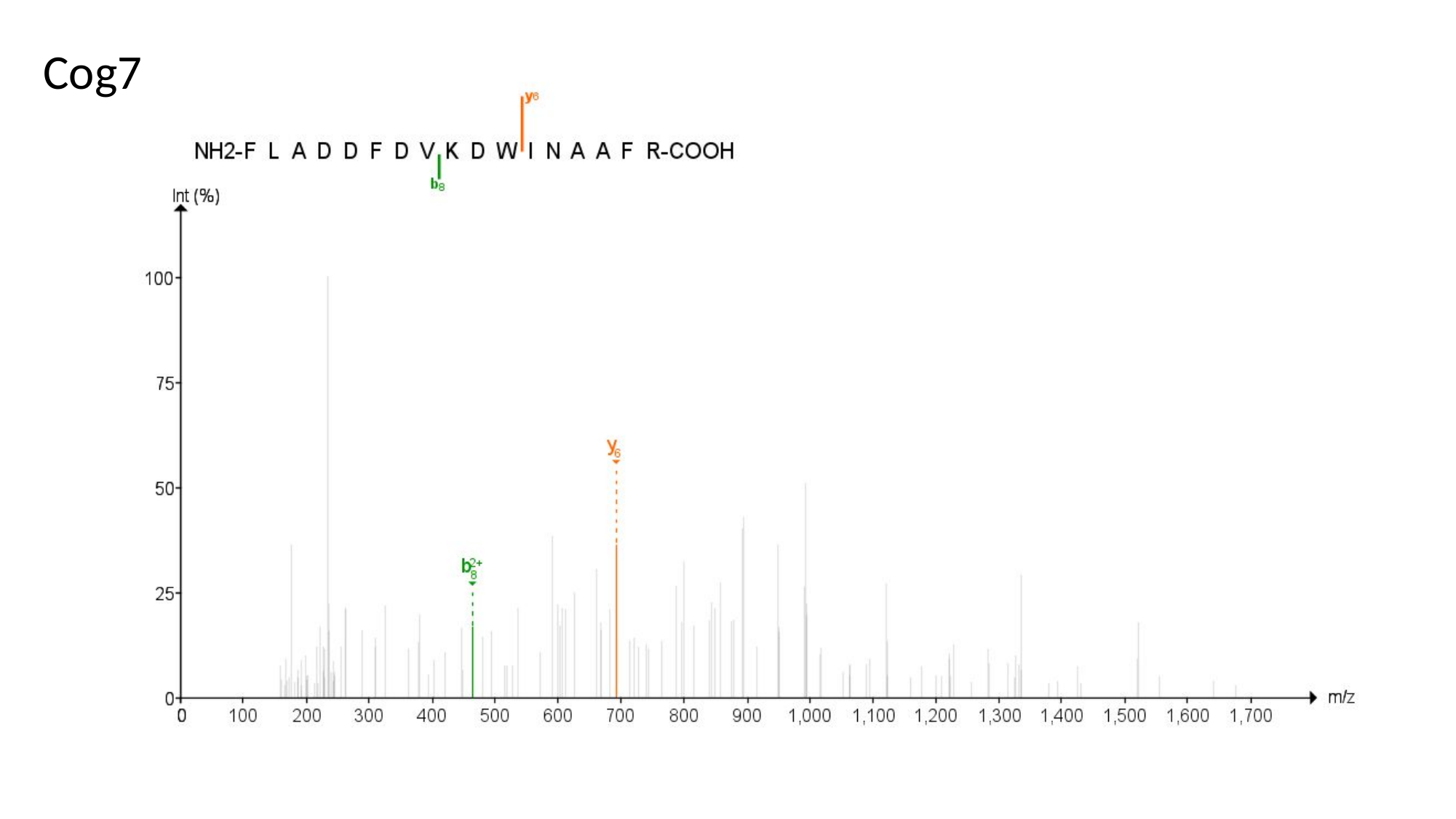

Cog7

## Slide 6
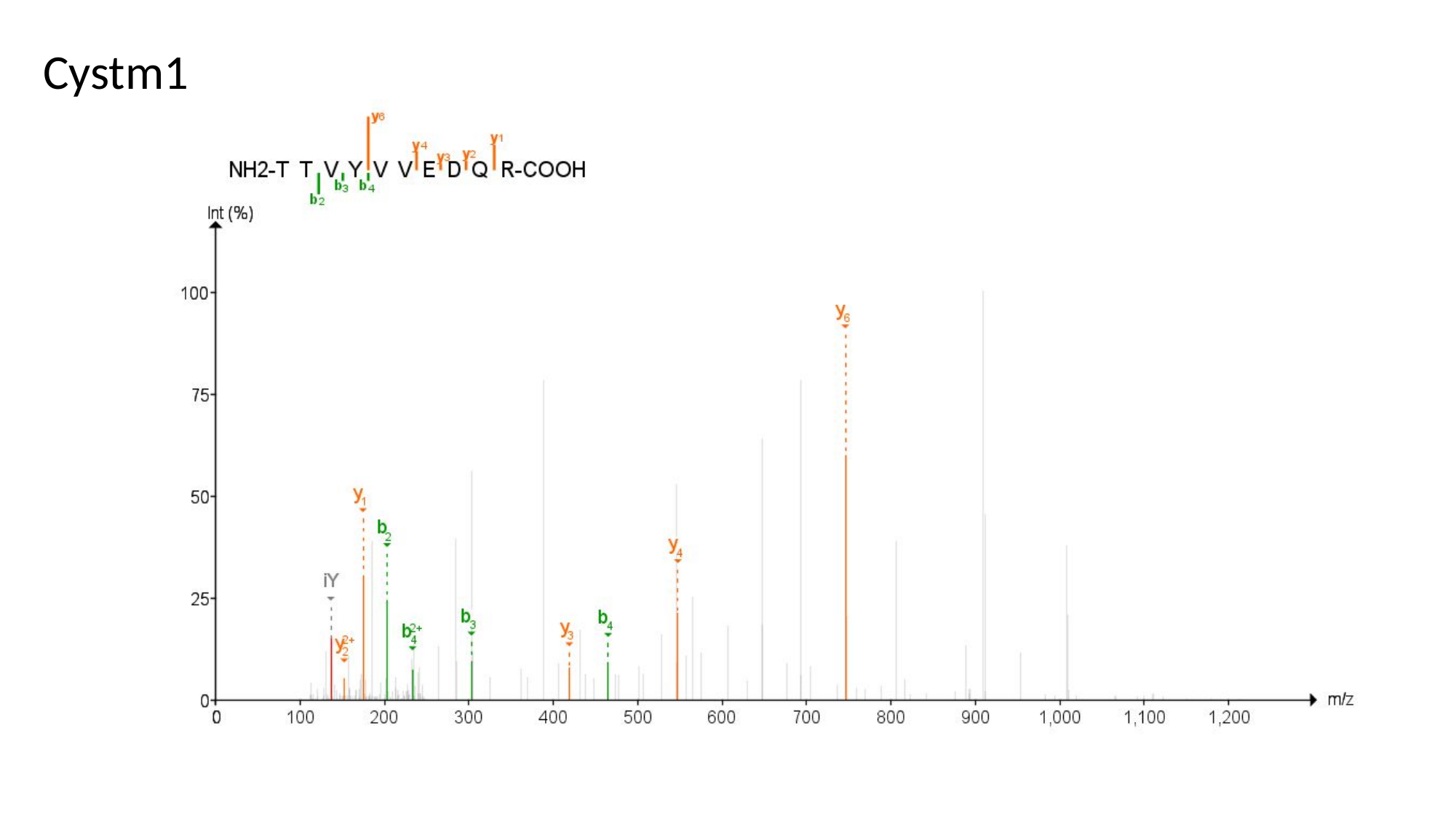

Cystm1

## Slide 7
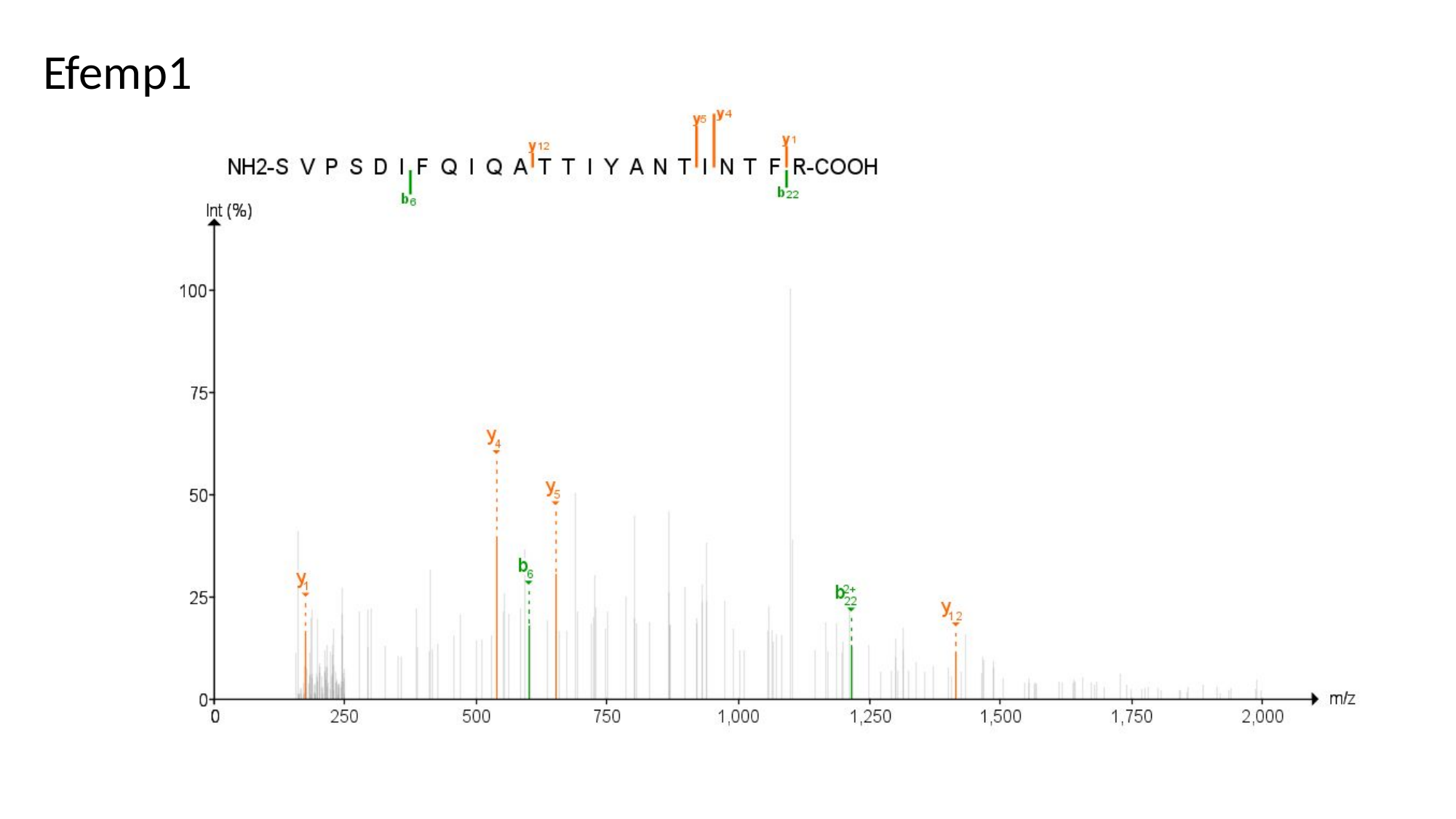

Efemp1

## Slide 8
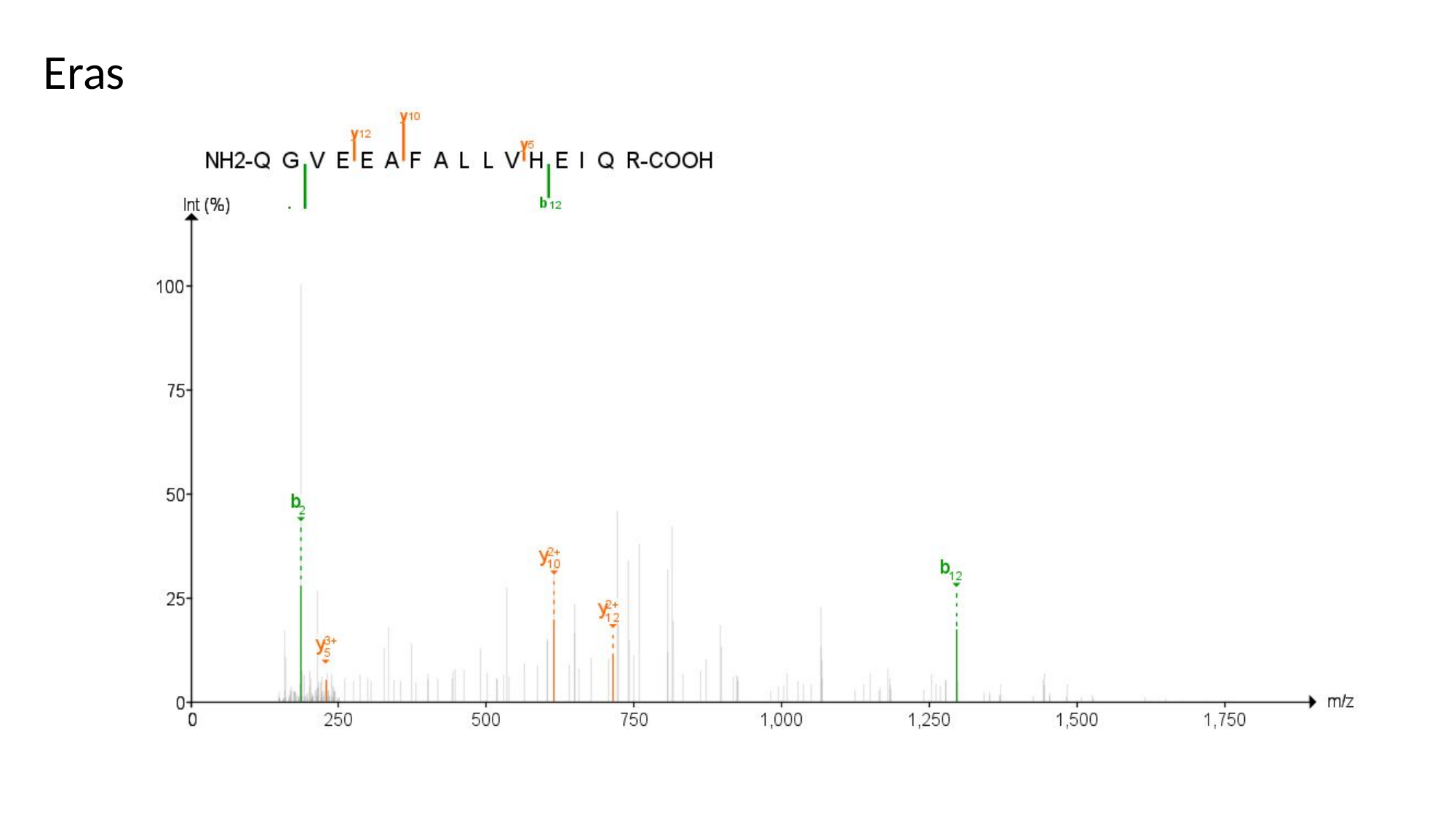

Eras

## Slide 9
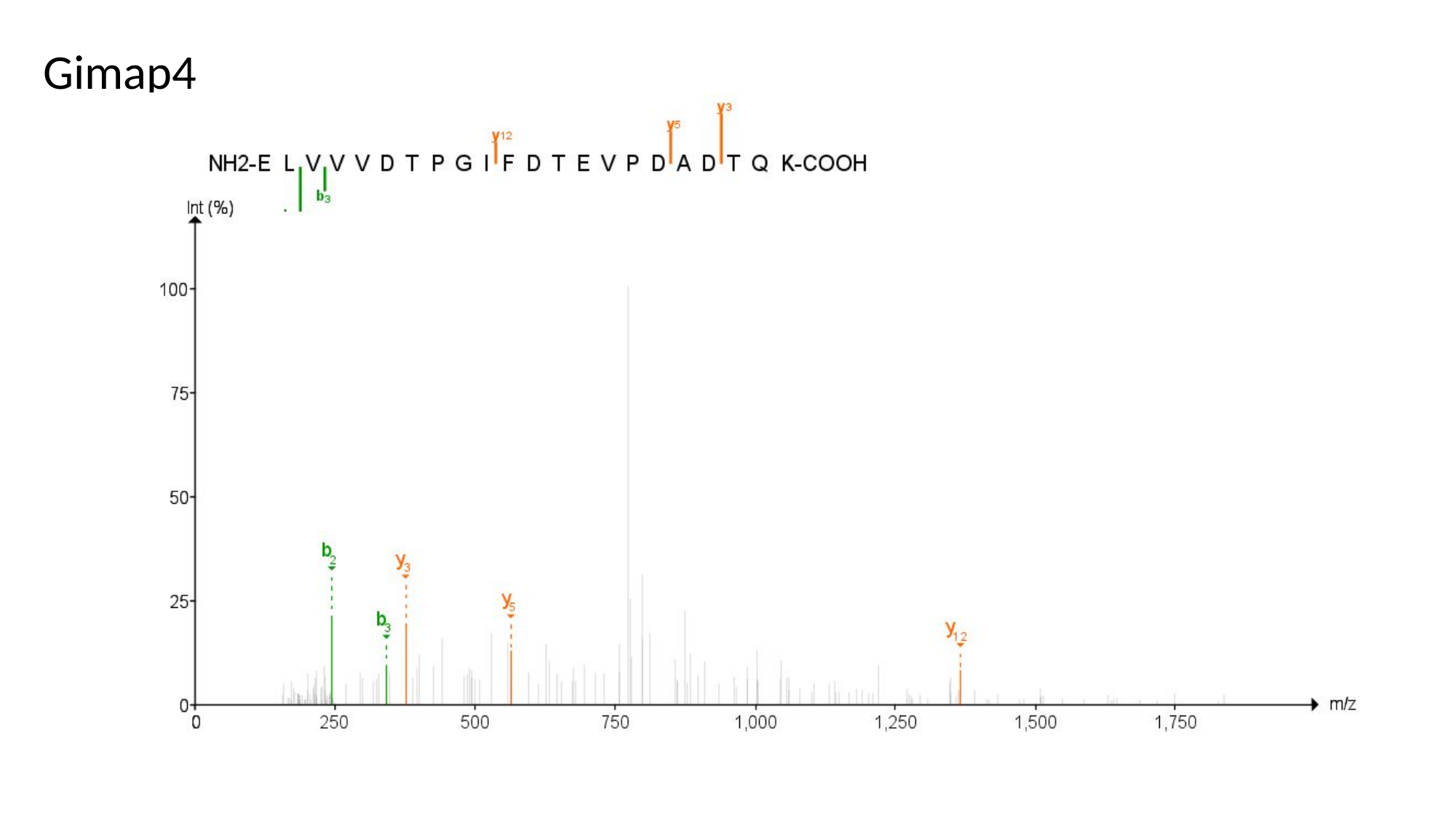

Gimap4

## Slide 10
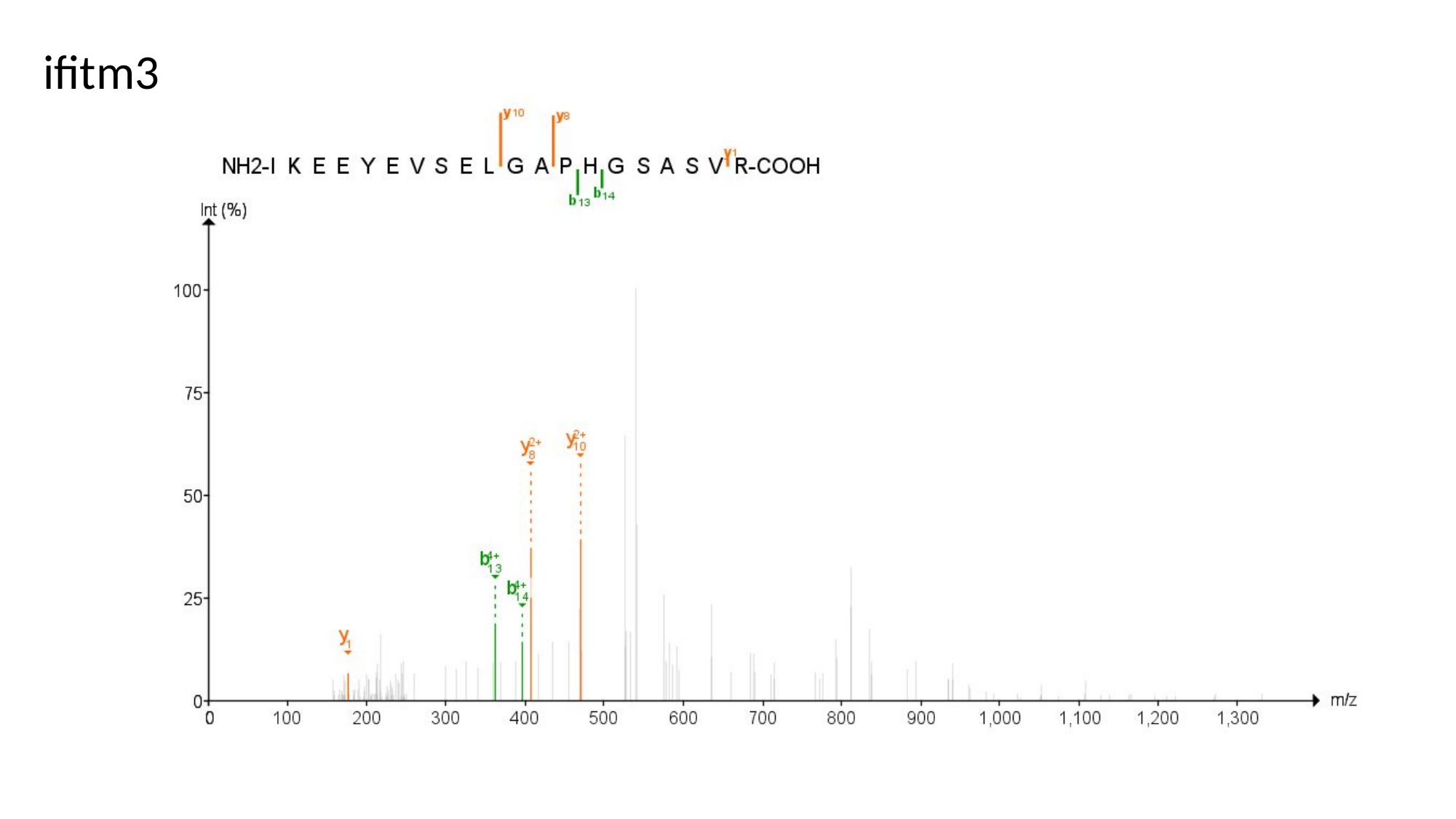

ifitm3

## Slide 11
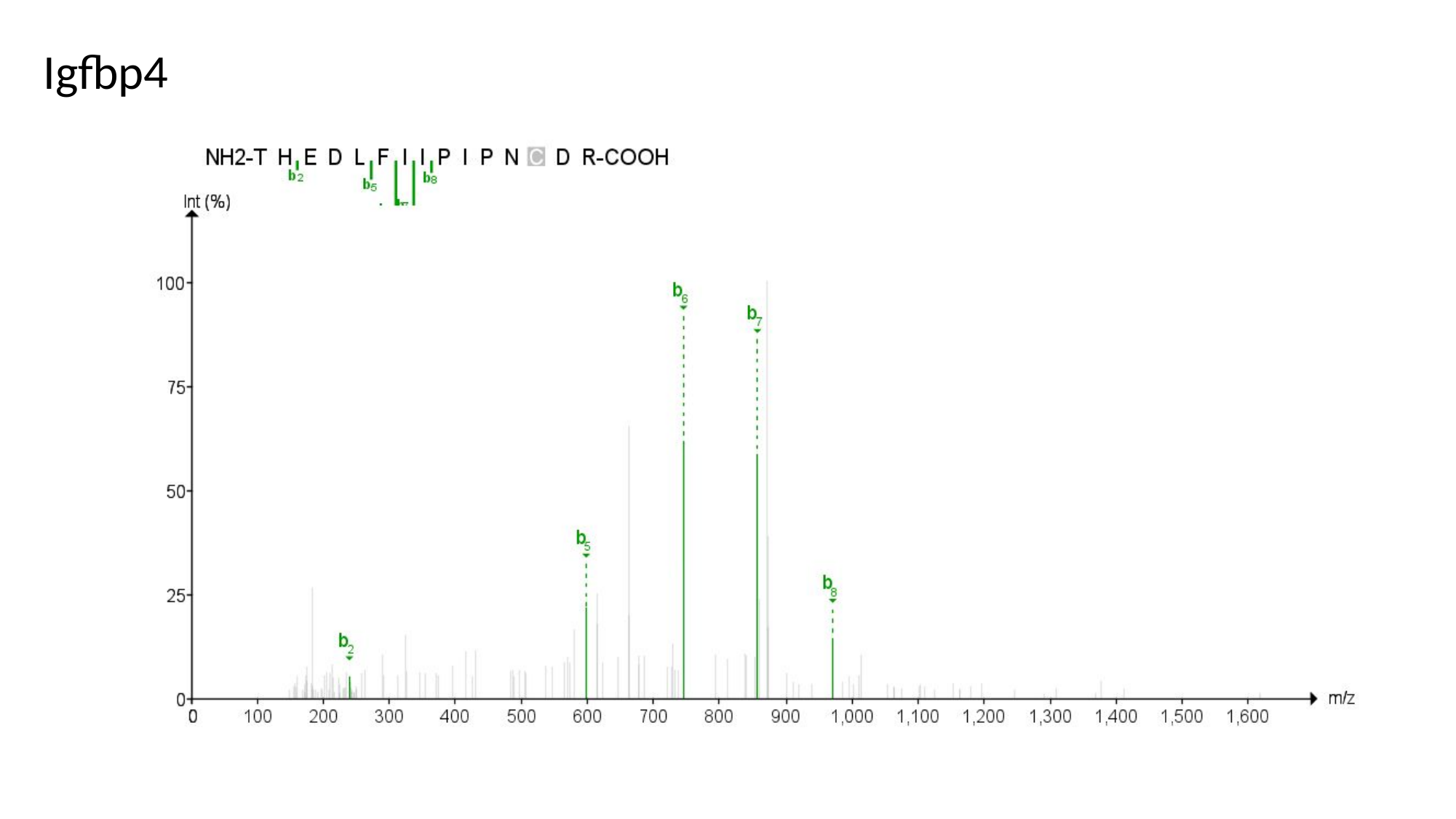

Igfbp4

## Slide 12
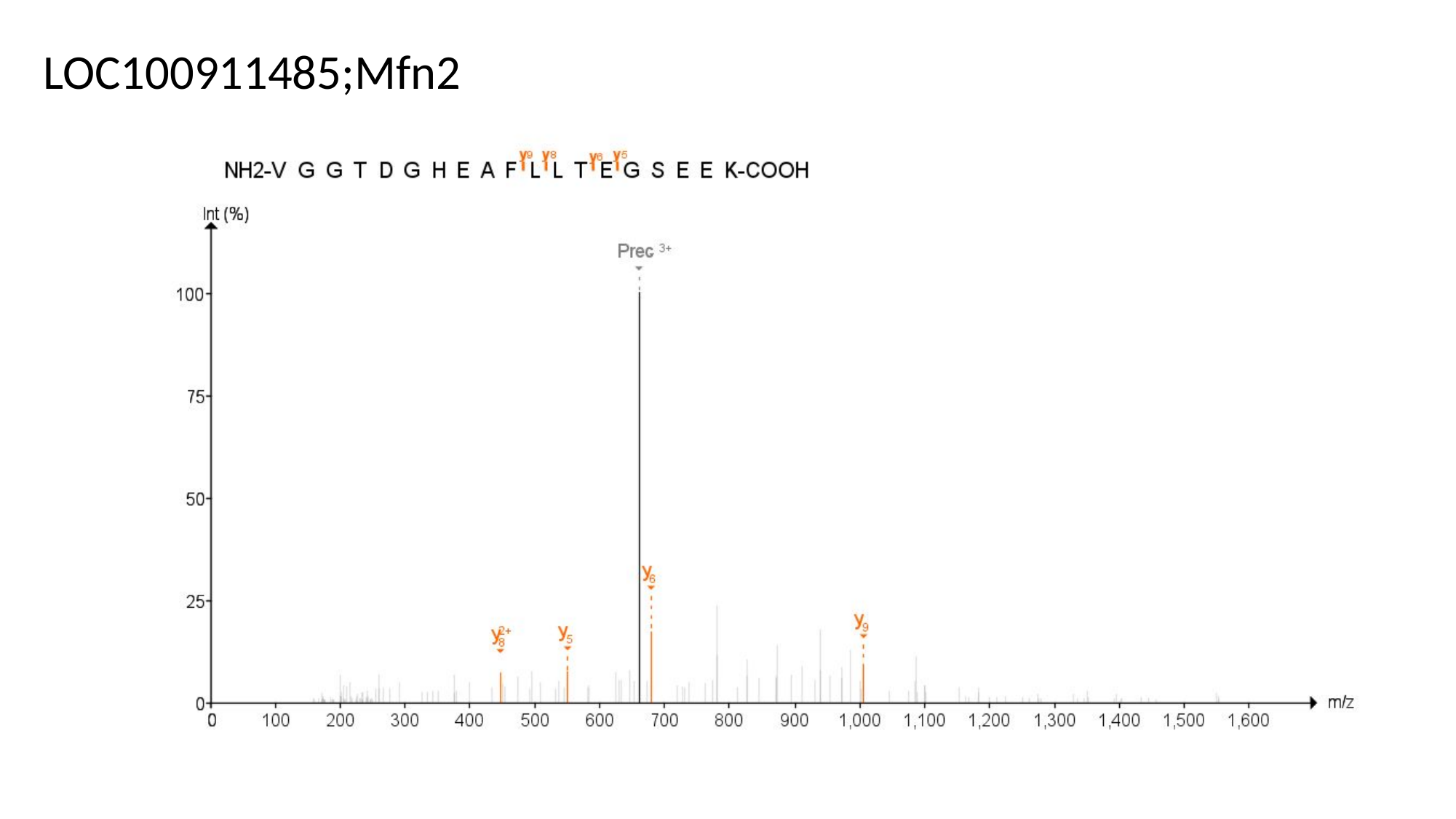

LOC100911485;Mfn2

## Slide 13
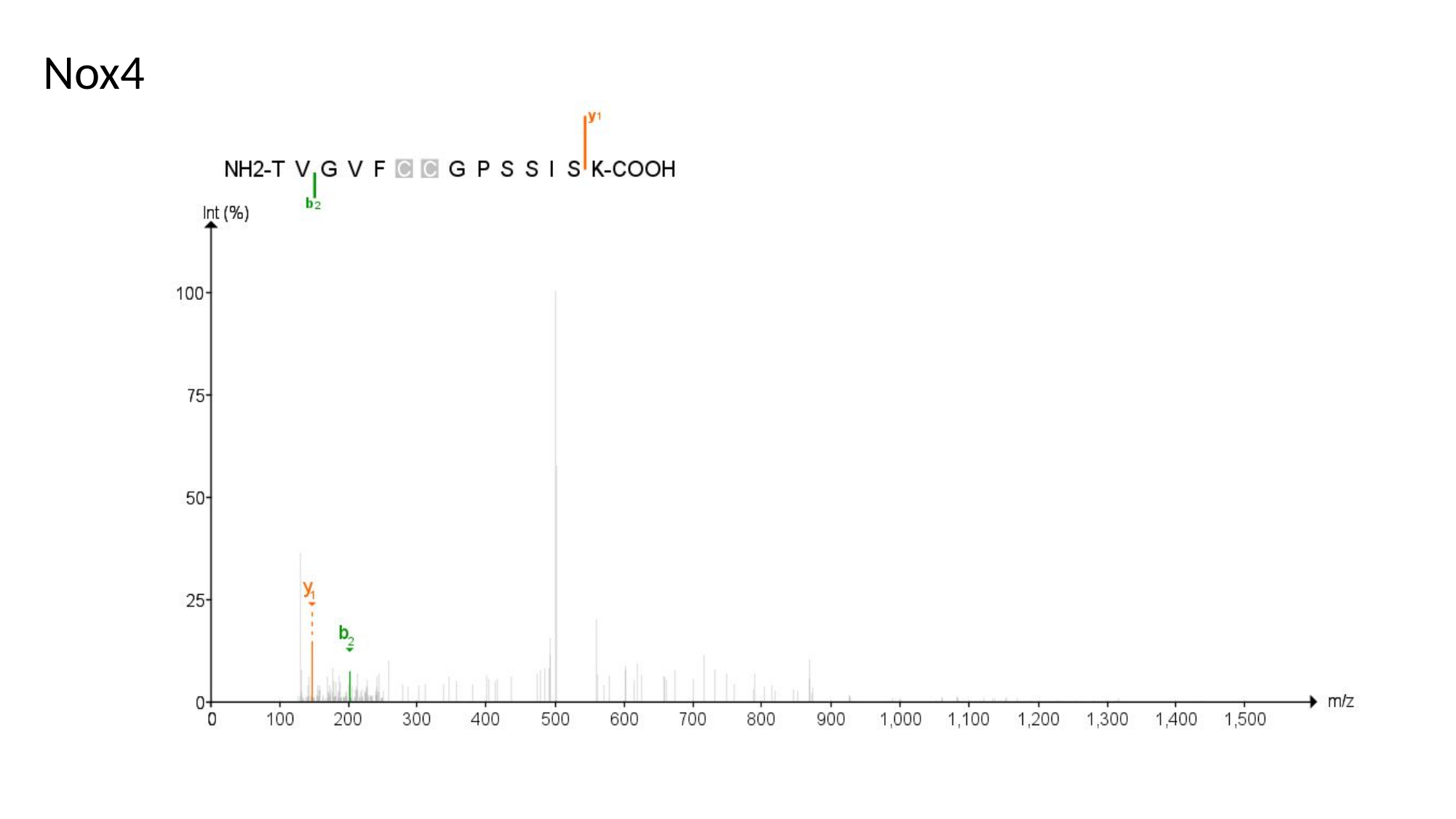

Nox4

## Slide 14
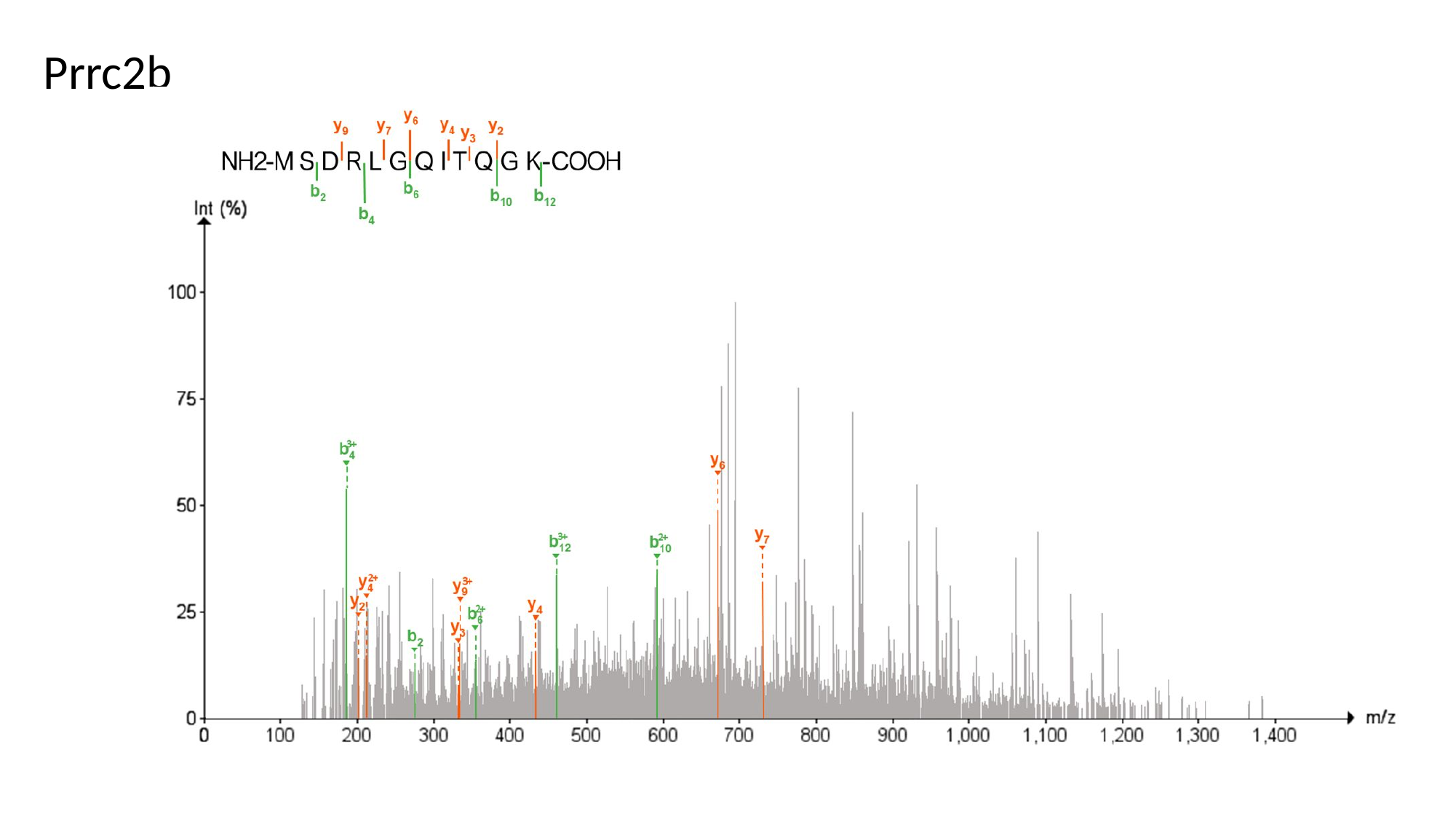

Prrc2b

## Slide 15
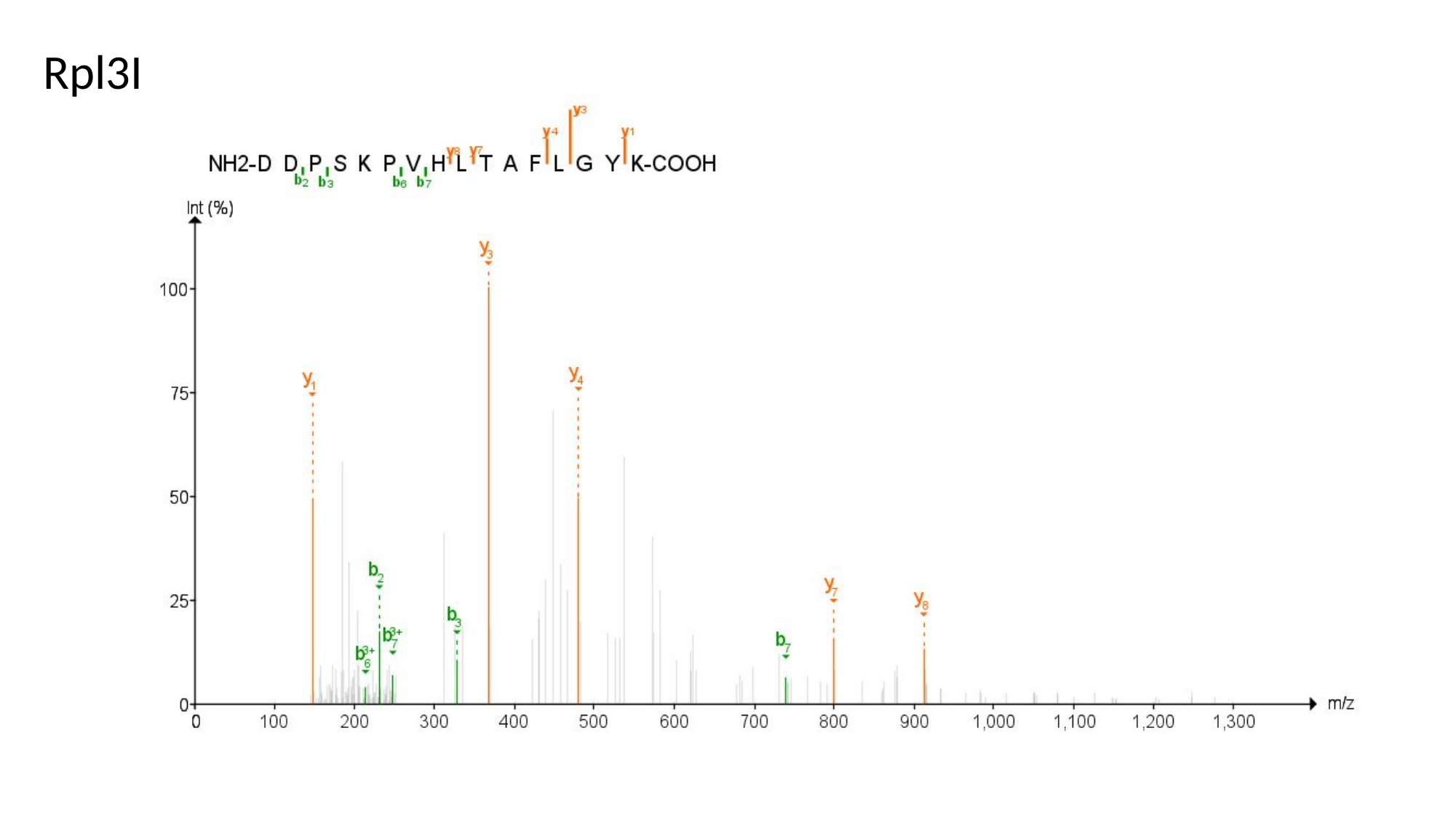

Rpl3I

## Slide 16
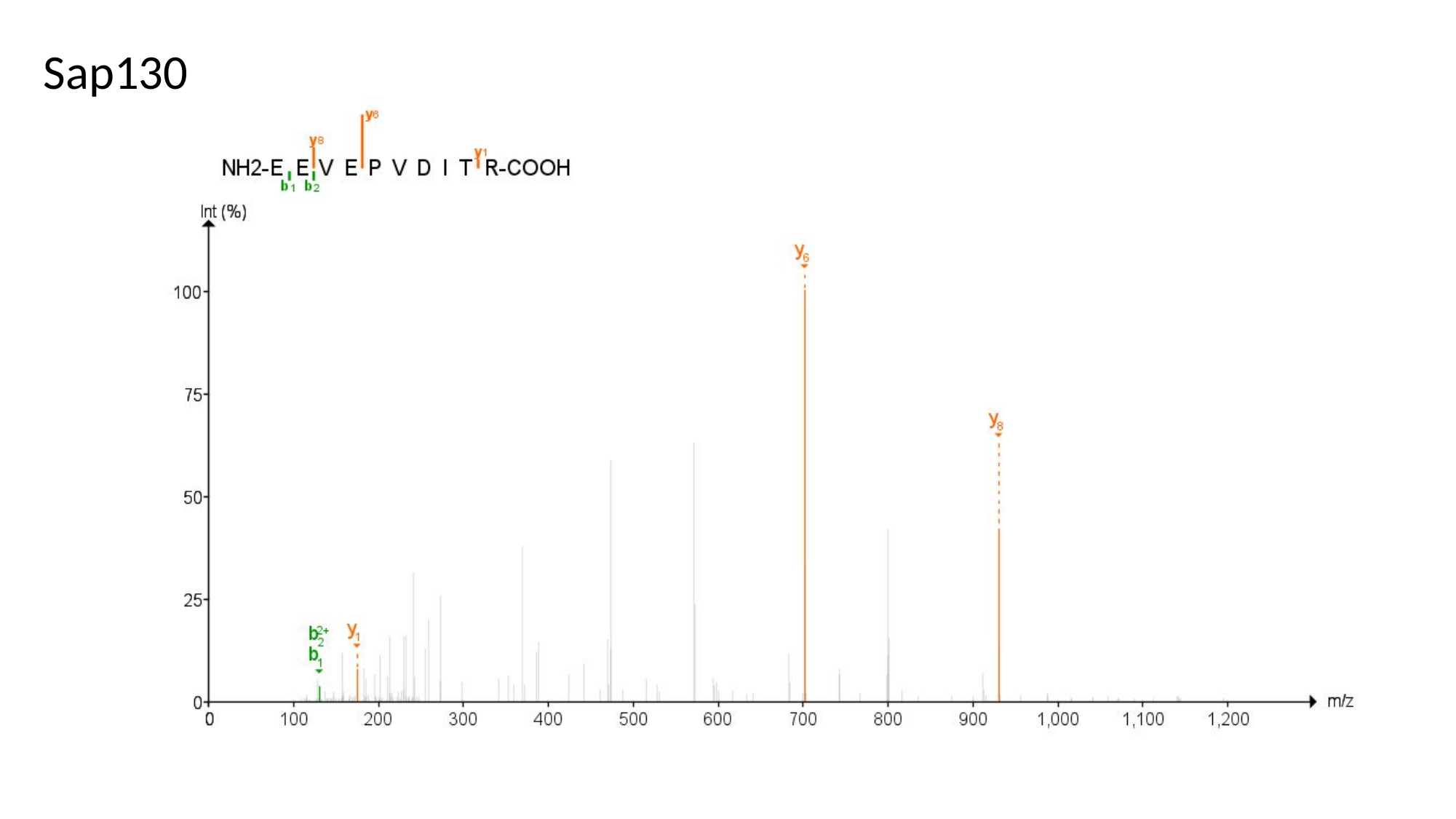

Sap130

## Slide 17
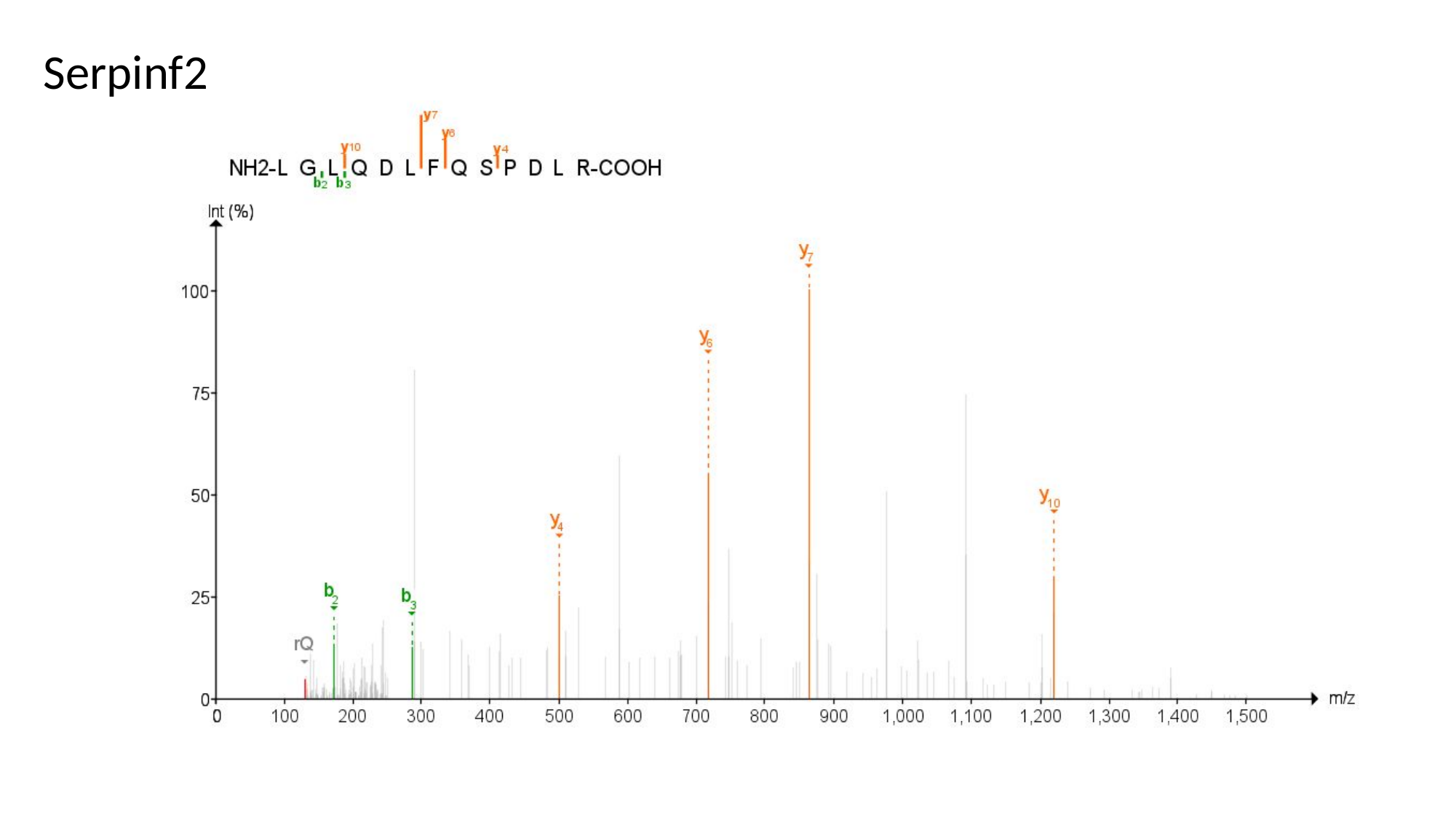

Serpinf2

## Slide 18
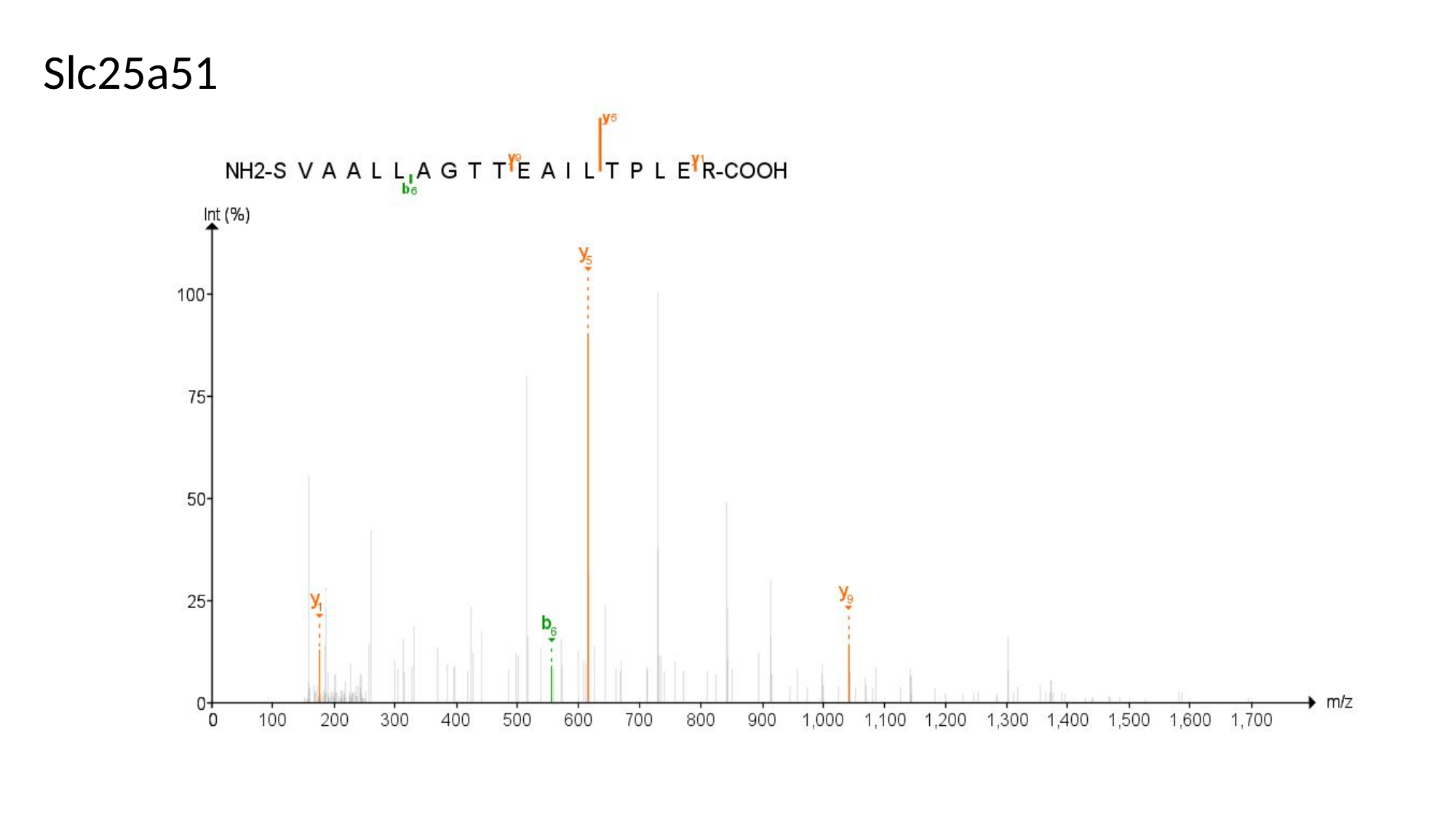

Slc25a51

## Slide 19
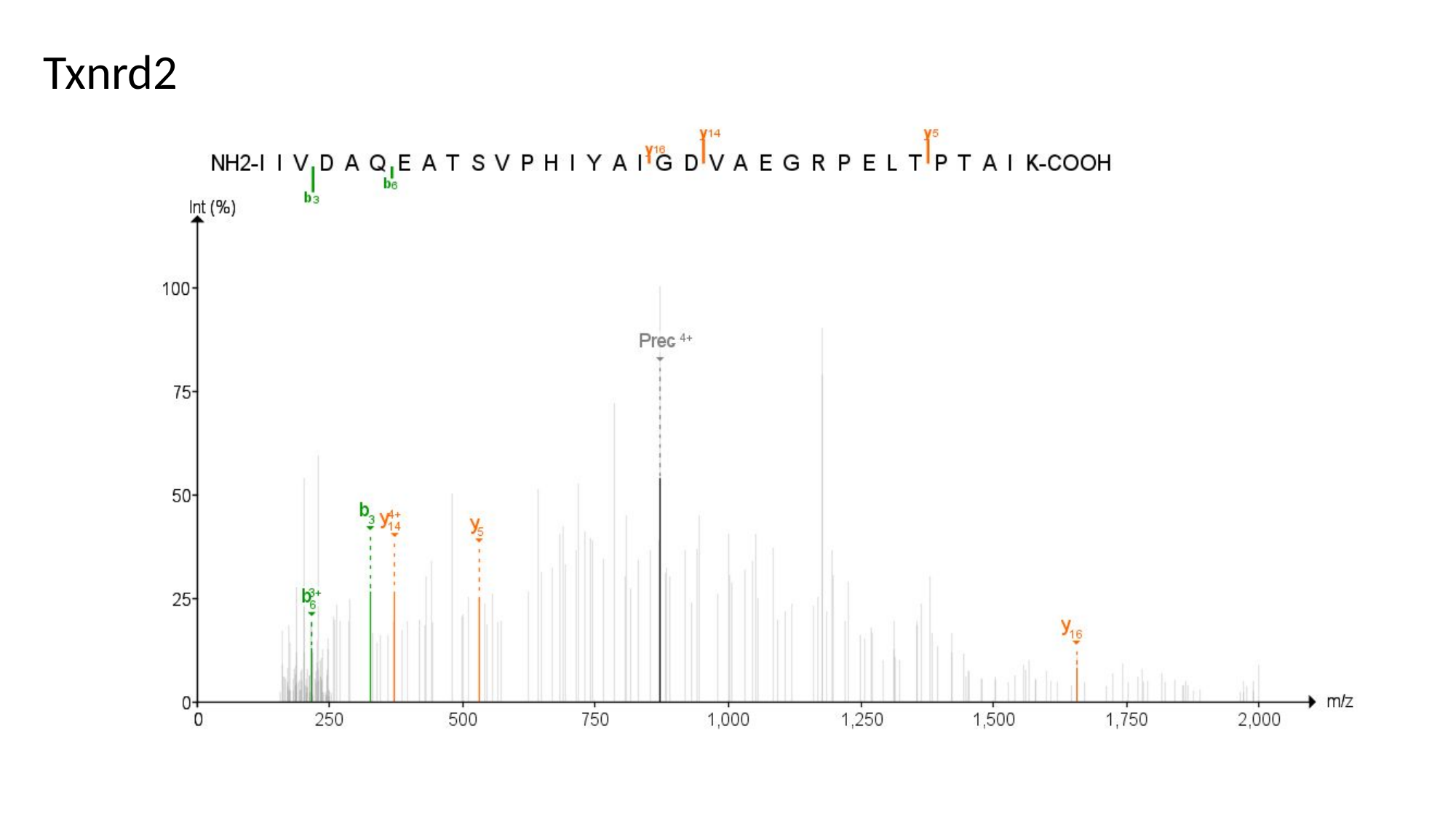

Txnrd2

## Slide 20
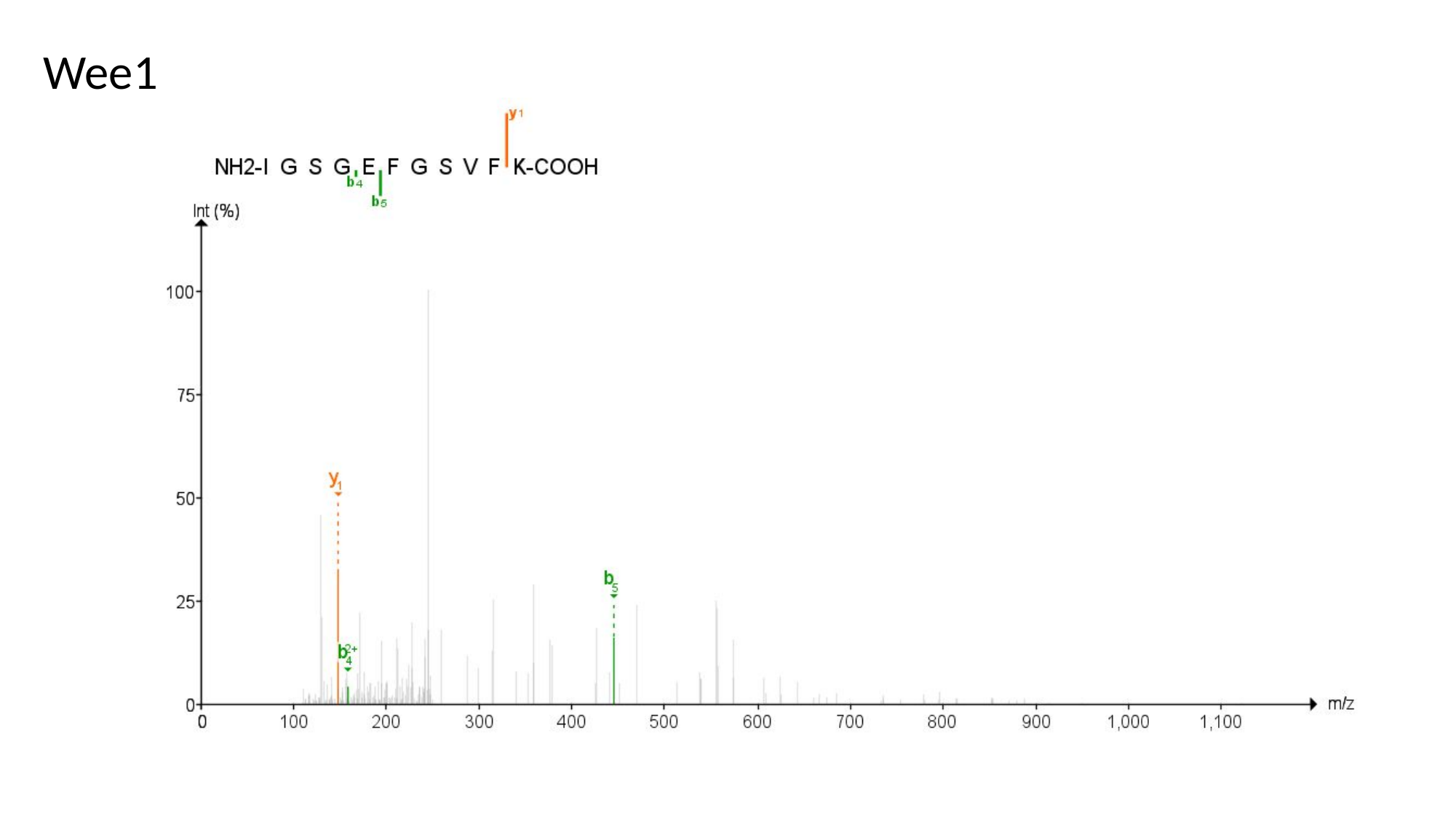

Wee1

## Slide 21
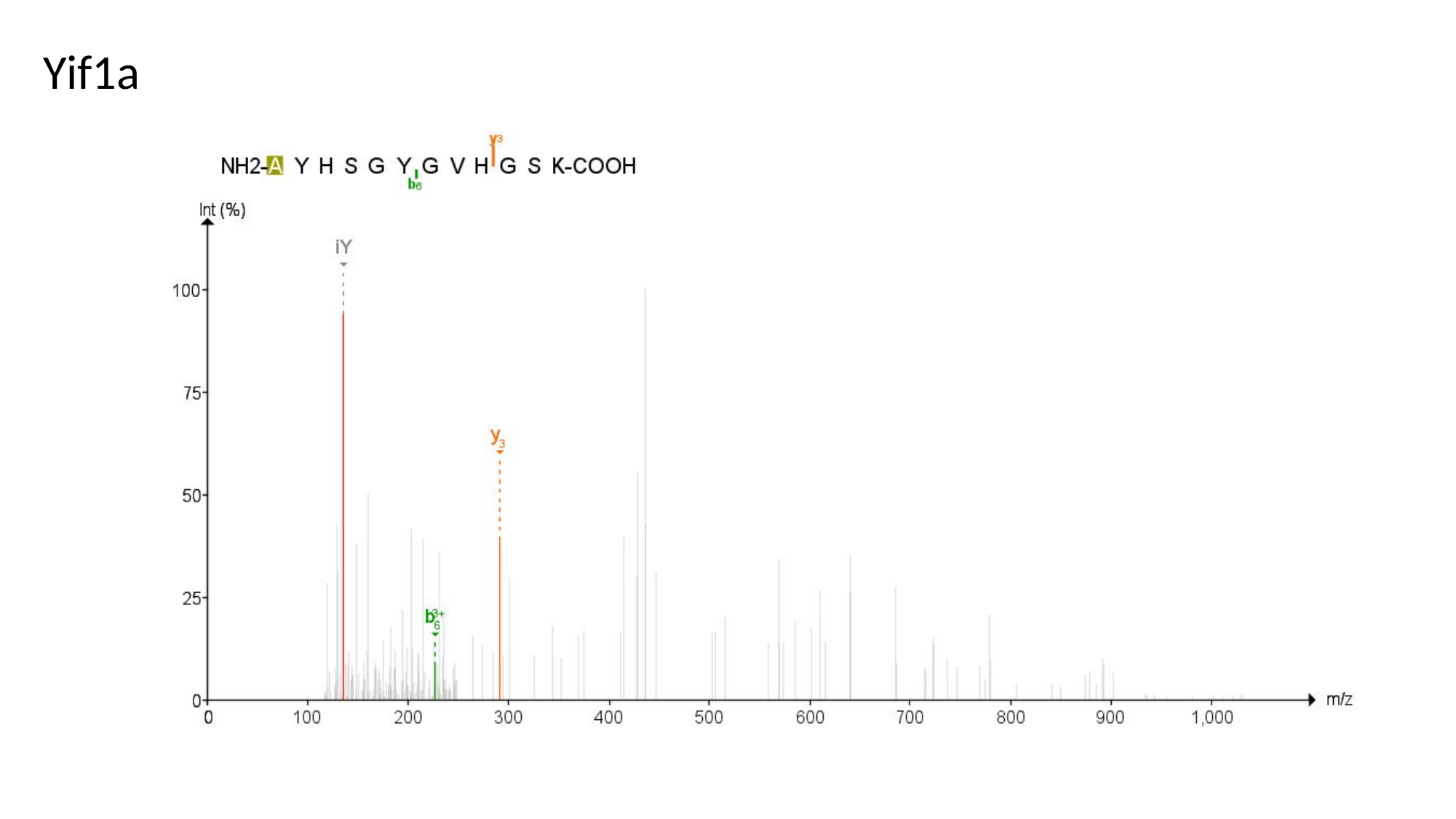

Yif1a

## Slide 22
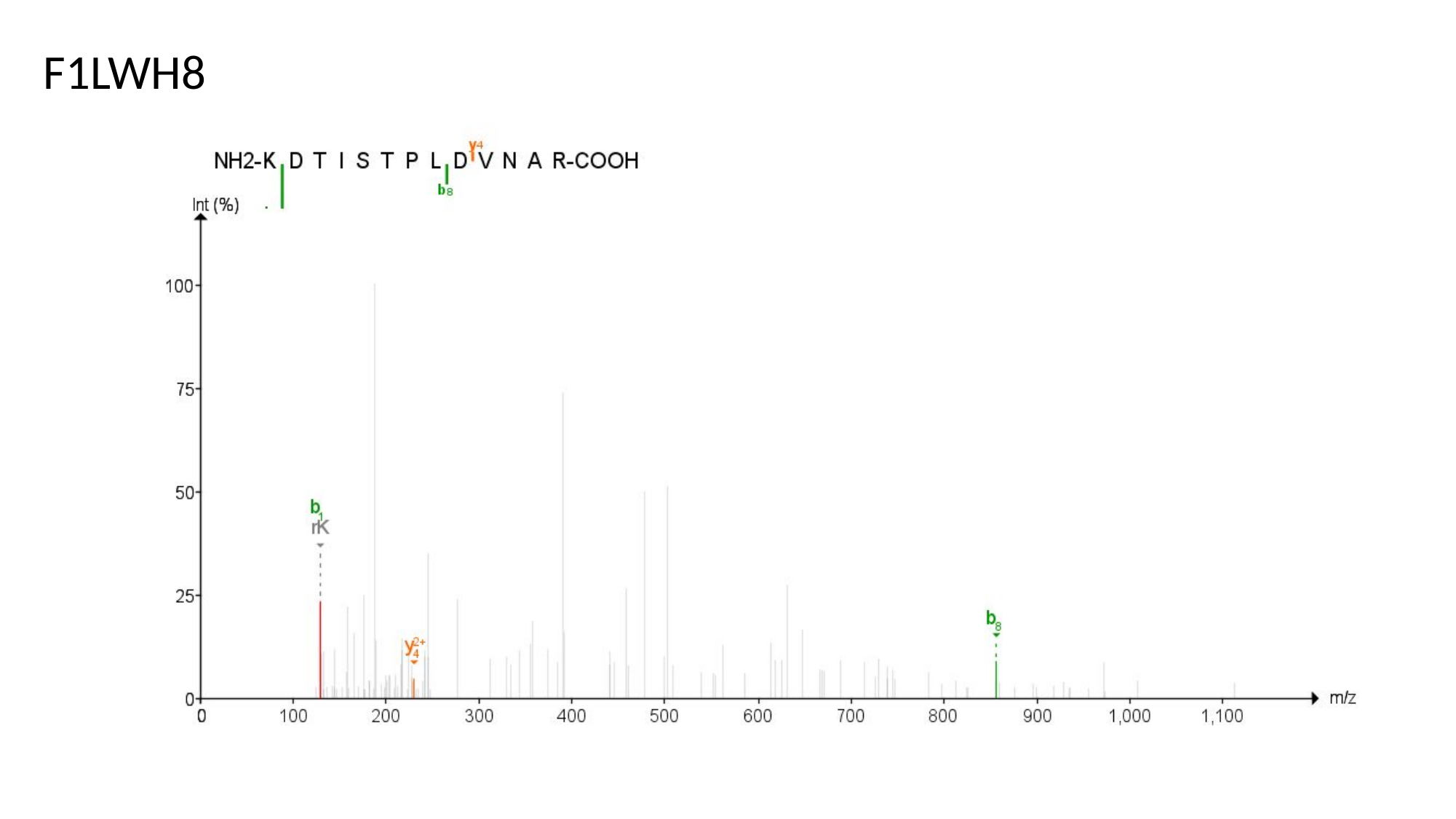

F1LWH8
